# Hierarchy Reproduction: Multiphasic Strategies for Tendon/Ligament–Bone Junction Repair

**DOI:** 10.34133/bmr.0132

**Published:** 2025-01-22

**Authors:** Kaiting Chen, Zezheng Liu, Xinying Zhou, Wanyu Zheng, He Cao, Zijian Yang, Zhengao Wang, Chengyun Ning, Qingchu Li, Huiyu Zhao

**Affiliations:** ^1^ Academy of Orthopedics, Guangdong Province, Orthopedic Hospital of Guangdong Province, The Third Affiliated Hospital of Southern Medical University, Guangzhou 510665, P. R. China.; ^2^ School of Pharmaceutical Sciences, Southern Medical University, Guangzhou, Guangdong 510515, P. R. China.; ^3^ School of Materials Science and Engineering, South China University of Technology, Guangzhou 510006, P. R. China.

## Abstract

Tendon/ligament–bone junctions (T/LBJs) are susceptible to damage during exercise, resulting in anterior cruciate ligament rupture or rotator cuff tear; however, their intricate hierarchical structure hinders self-regeneration. Multiphasic strategies have been explored to fuel heterogeneous tissue regeneration and integration. This review summarizes current multiphasic approaches for rejuvenating functional gradients in T/LBJ healing. Synthetic, natural, and organism-derived materials are available for in vivo validation. Both discrete and gradient layouts serve as sources of inspiration for organizing specific cues, based on the theories of biomaterial topology, biochemistry, mechanobiology, and in situ delivery therapy, which form interconnected network within the design. Novel engineering can be constructed by electrospinning, 3-dimensional printing, bioprinting, textiling, and other techniques. Despite these efforts being limited at present stage, multiphasic scaffolds show great potential for precise reproduction of native T/LBJs and offer promising solutions for clinical dilemmas.

## Introduction

Tendon/ligament–bone junction (T/LBJ) is an interface that facilitates the attachment of tendons or ligaments to bone, also known as enthesis. In sports medicine, injuries at the T/LBJ mainly manifest as anterior cruciate ligament (ACL) ruptures and rotator cuff (RC) tears. ACL rupture poses a challenge clinically and is frequently observed in football, skiing, basketball, and rugby players; its incidence among male athletes stands at 0.9 per 10,000 while being 1.7 times higher among female athletes [1]. In the United States alone, more than 200,000 individuals undergo surgical interventions annually for RC tears [2,3]. Unfortunately, retears commonly occurs within 6 months after surgery. Surgical failure rate even increases to over 90% as reported in massive or full-thickness RC tears [4–6], thereby imposing additional financial burdens on patients while exacerbating their pain.

As the terminal unit of the skeletal–muscular (tendon) and bone–bone (ligament) connection, T/LBJ withstands dramatic transformations in multiple dimensions, in order to maintain physiological homeostasis. For example, mineralization degree increases progressively from tendons/ligaments to bones. The arrangement of the extracellular matrix (ECM) exhibits histological changes from align to random. From a biomechanical point of view, tendons/ligaments adapt to a tensile environment, while the other ends approaching bones need to cope with the challenge of shear forces. These heterogeneous gradients in T/LBJ are tough to self-regenerate after being injured. In pathological conditions, the insertion site would establish disordered synovial tissue rather than an orderly reconnection. Although surgical repairment such as autologous transplantation can facilitate reattachment between osteotendinous stumps, achieving accurate healing remains challenging [7,8]. Therefore, it is necessary to reconstruct the T/LBJ gradient microenvironment to provide precise guidance for stem cell differentiation.

To tackle this obstacle, innovative strategies represented by multiphase engineering have invigorated studies on T/LBJ healing. Scaffolds, patches, or hydrogels incorporate diverse biophysical and biochemical cues to achieve distinct biomimetic features that meet the specific needs of target tissues. The focus lies in regulating the repair process that necessitates fundamental cellular activities, directed differentiation of stem cells, and orderly ECM deposition [9], ultimately leading to physiological restoration. These designs also fulfill the fundamental needs of interface tissue engineering, including facilitating nutrient permeation, promoting microenvironment crosstalk, and enabling seamless tissue integration. Thus, multiphasic strategies exhibit potentials to provide novel protocols for clinical practice.

**Fig. 1.**
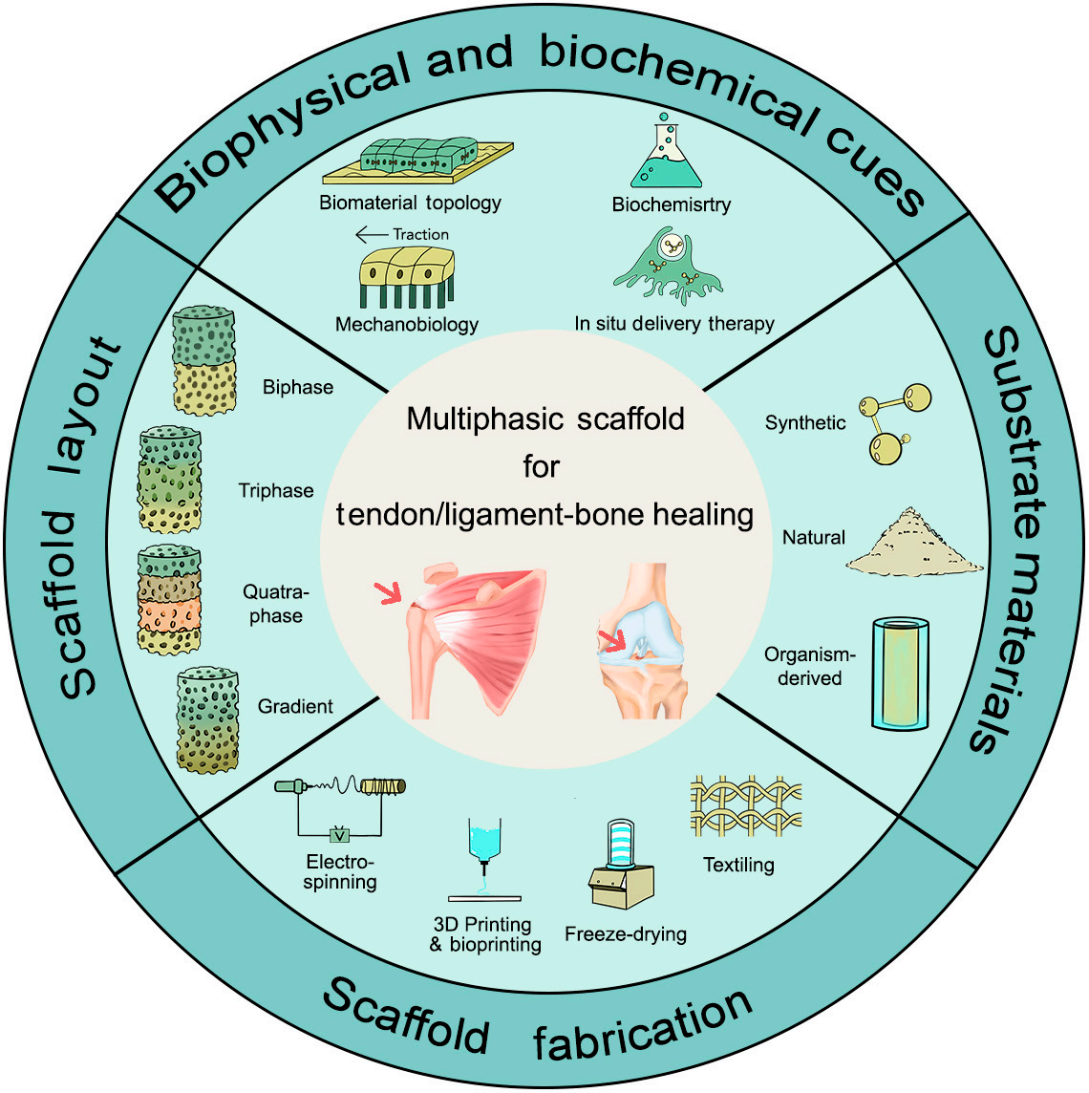
Schematic illustration of various strategies in multiphasic engineering to accelerate T/LBJ healing.

In this review, we first delve into the physiological hierarchies and healing process of T/LBJ. Second, we introduce basic information about multiphase engineering from the stand points of substrate materials and scaffold layouts. Notably, the basis of provided cues is categorized into 4 groups: biomaterial topology, biochemistry, mechanobiology, and in situ delivery therapy. Their applications are further elucidated with an extra part to introduce their interaction network. Subsequently, various fabrication methods are consolidated to address the limitations of existing clinical treatments by collating past studies. Finally, current shortcomings and advanced strategies are proposed as references for subsequent researches. The main context of this review is summarized in Fig. 1.

## Overview of T/LBJ

### T/LBJ hierarchies

**Fig. 2.**
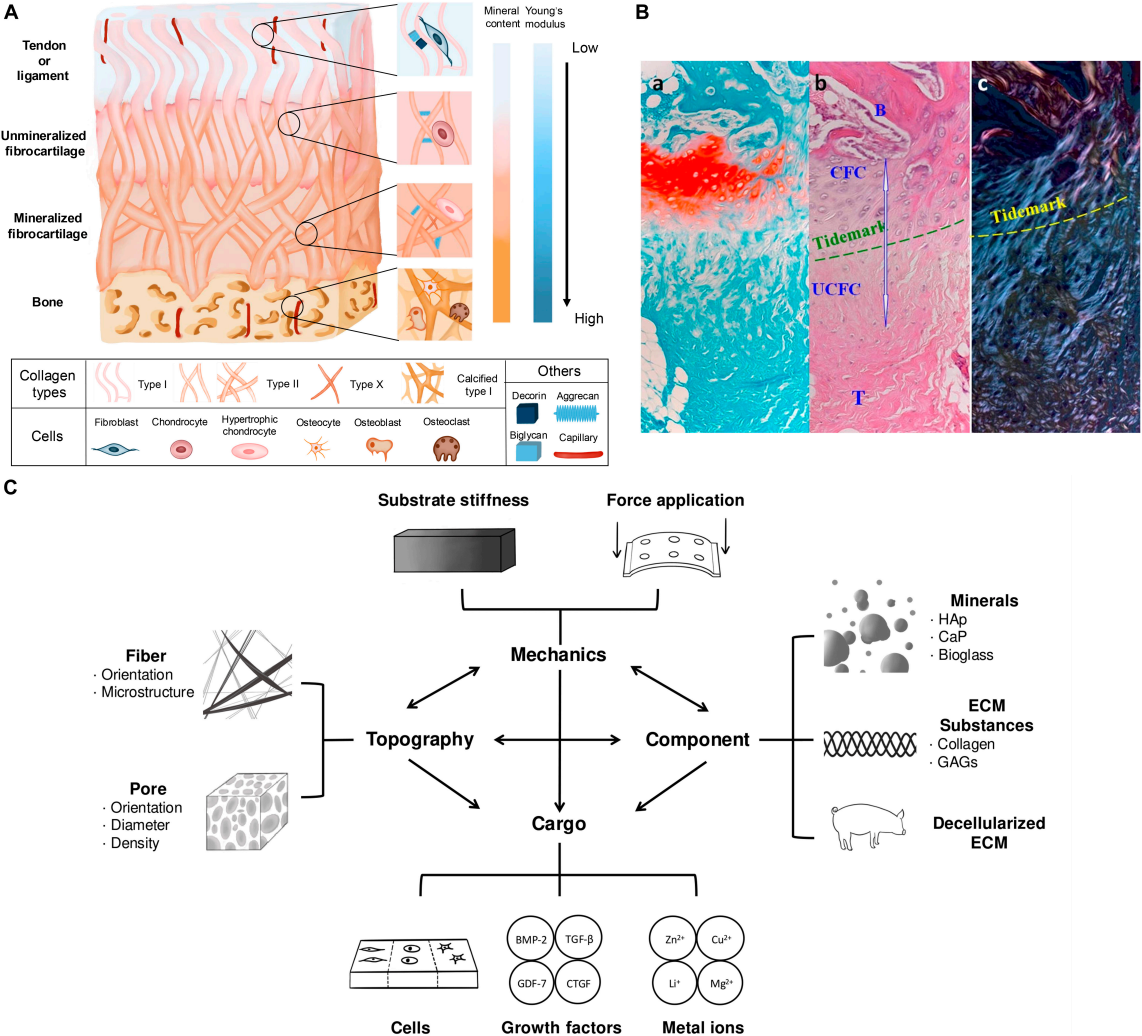
Overview of physiological tendon/ligament-to-bone hierarchy and the multiphasic strategies for healing inspired by this. (A) Schematic illustration of the tendon/ligament–bone interface and zoomed-in graph manifesting microstructure and biological members in quadruple region. The ascending trend of mineralization and stiffness from tendon/ligament to bone is displayed aside. (B) (a) Safranin-O staining, (b) hematoxylin and eosin (H&E) staining, and (c) polarized microscopic images of the physiological 4-layer structure of enthesis, and the gradual transition and tidemark should be emphasized. Magnification, 20×. B, bone; CFC, calcified fibrocartilage; UFC, uncalcified fibrocartilage; T, tendon. Reproduced with permission [251]. Copyright 2010, Springer Nature. (C) Macroscopic view of recent efforts to reproduce tendon/ligament-to-bone hierarchy. Specific auxiliary cues of the 4 aspects are listed beside. The interconnected network of 4 categories is depicted in the center of the graph (2-way arrows representing mutual impact and one-way arrows indicating unilateral influence).

T/LBJ is representative of interface tissues, encompassing tendons, fibrocartilages, and bones. The classical quadruple structure is merely an artificial division because the physiological microenvironment gradually transitions and the boundaries are blur. In summary, distinctions can be classified into 4 aspects: ECM topography, composition, mechanical properties, and histological performance [10,11], as presented in Fig. 2A and B.

Tendon is a strong, inelastic connective tissue that facilitates the transmission of muscle force. The slender tenocytes that scattered among fibers belong to fibroblast (FB). Plus, tendon stem/progenitor cells (TSPCs) engage tendon homeostasis and repair, but the preservation of osteogenic potential allows for heterotopic ossification in the complex traumatic environment [12]. FBs and fibrochondrocytes secrete fibrils (nanoscale), which are gradually assembled into fibers (micrometer-scale), fascicles (micrometer-scale to milli-scale), tertiary fiber bundles (milli-scale), and finally strip-like tendons, which are internally parallelized [6,13]. Although type I collagen (Col I) makes up the vast majority of tendon fibers, the role of Col III, which accounts for 1% to 1.5%, in regulating the diameter of Col I fibers has been reported [14]. Glycoproteins and proteoglycans participate in fiber network construction and surface chemical modification. In terms of the inorganic phase of ECM, mineral content undergoes a sharp decrease from bone to tendon. During the deposition process, combination of the amorphous calcium phosphate (CaP) (with irregular crystal structure) and amino acid residues of collagen fibers was performed through electric charge, which in turn is converted to hydroxyapatite (HAp) [15]. The discontinuous formation of CaP crystal structure is the main reason for macroscopic mineralization gradient.

Fibrocartilage is a flexible connective tissue providing cushioning and serves as a site for tendon attachment, which can be further categorized into mineralized and unmineralized zones, with a basophilic tidemark labeling in between (Fig. 2B) [16]. Hypertrophic chondrocytes at calcified fibrocartilage tend to be round, while chondrocytes in noncalcified areas exhibit oval shape [17]. Unlike bones and tendons, Col II dominates cartilage ECM. Moreover, mineralized fibrocartilage contains notable amounts of type X collagen, differing from nonmineralized one [15]. Also uniquely, capillaries do not penetrate either type of cartilage. This avascular nature results in a poor regenerative response, leading to limited cell migration and matrix synthesis upon injury. Lacunae (hollows to embed chondrocytes) serve as characteristic microstructure in cartilage region, and changes in pore orientation with location are observed [18]. Biomechanically speaking, cartilage plays a pivotal role in the T/LBJ, acting as a smooth and resilient interface that transitions from the soft tendon to the hard bone, providing shock absorption and maintaining joint integrity.

Bone is a rigid connective tissue that provides structural support. Bones are mainly distributed with osteocytes (mature cells), osteoblasts (precursor cells), and monocyte-derived osteoclasts, which regulate the dynamic equilibrium of lysis and reconstruction. In bone tissue, HAp accounts for about 50% of the volume and 69% of the dry weight [15,19]. Another important ECM component is calcified Col I. As a porous structure, the pore size of bone increases from 10 to 50 μm (cortical bone) to 300 to 600 μm (cancellous bone). Microimaging has led to the discovery of micro- and nanoscale porous bone hierarchies such as Volkmann canals and canaliculi [20,21]. In terms of fiber connectivity, the fiber deviation angle increases and fibers gradually stagger approaching bone. The end of entheses dispersedly inserts into bone [22], which eventually orient randomly to gain improved compression strength. As a consequence of these, mechanical tests revealed a significant increase in elasticity modulus from tendon (0.1 to 1 GPa) to bone (10 to 20 GPa) [23–25]. The progressive connection prevents misalignment between tendons and bones, which attenuated shear strength to prevent stress overload.

### Healing process and clinical limitation of T/LBJ

Generally, tendon/ligament–bone healing is initiated by inflammatory response. Following injury, necrotic or apoptotic marginal cells and tissue debris are generated, leading to enhanced influx of immune cells (especially macrophages) through a crosstalk network, which involves increased capillary permeability [26], phagocyte clearance, and activation of inflammatory factor release. Matrix metalloproteinases (MMPs), as representative proteolytic enzymes, degrade collagen to initiate ECM remodeling [27]. The hydrostatic equilibrium of the joints is disrupted, and the effusion in the joint cavity increases. Meanwhile, synovial fluid biochemical properties, such as viscosity and protein concentration, hinder joint lubrication and nutrient supply. These result in a pathological microenvironment similar to that of osteoarthritis [28]. Synovial-like disordered connective tissues would be formed, which interfere with the reconnection of tendon/ligament–bone insertion and further induce pain and bone destruction.

For ACL ruptures, the best surgical treatment in sports medicine is ligament reconstruction (autologous/artificial graft combined with interfacial screw fixation). For RC tears, the surgeon stitches the severed ends of the tendon to each other or to the bone under arthroscopic, depending on the tear type. Massive RC tears involve autologous tendon grafting or even shoulder joint replacement. The postoperative regeneration process is consequently unstable, and the joint flexibility could not return to pre-injury state, which is often unacceptable for athletes. The clinical dilemma sources from the lack of regenerative niche. The tendon/ligament–bone interfaces formed by the graft or residual tissue in the bone tunnel are histologically mainly fibrovascular scars [29] and gradually turn to vertical collagen fibers attaching to the bone, called Sharpey-like fibers, rather than the original 4-layer transition structure [30,31]. The graft integrates into the surrounding bone 1 year postoperatively but fails to exhibit an orderly hierarchy of collagen fibers and mineral distribution [32]. This weakens the original mechanical properties, hampering long-term clinical efficacy. Additionally, if inflammation becomes chronic or pathogenic factors persist long-term, complications will arise. RC traction failure can initiate degenerative pathologies including amyotrophy, fibrosis, and adipogenesis [33–35], leading to continuous pain and disability. After ligament rupture, stability fails to be maintained and adnexal structures such as meniscus, muscles, and articular cartilage may develop chronic osteoarthritis [36].

## Basic Designs

### Substrate materials

Substrate materials directly interact with cells, providing the skeleton for tissue healing and microenvironment remodeling. Certain characteristics are necessary to permit basic biological activities such as adhesion, migration, growth, proliferation, and secretion [37]. Principles are as follows: (a) biocompatibility, avoiding hazardous substances in both the material and production process; (b) strong mechanical strength, providing essential support and effective bioforce conduction; (c) biodegradability, capable of decomposition in the body, and decomposition products should align with the first 2 principles; (d) machinability, moldable into specific shapes and/or compatible with specific substances for establishing gradients. Additionally, some designs require more stringent requirements, such as controllability, which means that they can be adjusted according to biomimetic needs. Optional materials can be categorized as synthetic, natural, or organism-derived according to their source. A variety of common materials are summarized in Table 1, listing their application scenarios, advantages, and disadvantages.

**Table 1.** Detailed summarization of common materials and relating information for tendon/ligament–bone hierarchical engineering. PCL, poly-ε-caprolactone; PLCL, poly(l-lactide-co-ε-caprolactone); PLA, polylactic acid; PLGA, poly(lactic-co-glycolic acid); SF, silk fibroin; GelMA, gelatin methacryloyl; dECM, decellularized extracellular matrices; RGD, Arg-Gly-Asp.

Type	Materials	Main construction method	Common application	Merits	Shortcomings	References
Synthetic polymer	PCL and PLA	Electrospinning; 3D printing; melt electrowriting	Bone phase; tendon phase	Biocompatible; low melting point; permitting various copolymerization and surface modification; adjustable biophysical properties	Low bio-affinity; inadequate mechanical properties; low degradation rate; producing acidic decomposition products	[97,252–256]
	PLCL and PLGA	Electrospinning; 3D printing	Bone phase; tendon phase	Biocompatible; programmable physical characteristics and biodegradability (by adjusting the proportion of copolymers)	Insufficient cell adhesion	[4–37,43,55,67,71,74,86,97,226,251–256]
Natural polymers	Collagen	Freeze-drying; bioprinting	Bone phase	High bio-affinity; rheologically excellent after modifications; consistent with natural tendon-to-bone ECM component	Poor mechanical properties; introducing biohazardous catalysts during crosslinking	[22,56,95,257]
	SF	Electrospinning; textiling; freeze-drying	Tendon phase	Good water solubility; outstanding elasticity and toughness	Poor mechanical properties; unobvious bio-affinity; immunogenicity; high processing requirements	[44,59,107,128,173,227]
	Gelatin and GelMA	Bioprinting	Bone phase	Abundant modifiable sites; possessing bioadhesive RGD sequences; suitable viscosity and printability with shear thinning	Poor mechanical properties; insufficient thermal stability	[116,121,163,165]
Organism-derived materials	dECM	Bioprinting	Bone phase; tendon phase	Preserving the structural and componential integrity of ECM; elimination of cellular remnants and antigenicity	High technical requirements; long pretreatment time	[46,50,136,137,143]

### Scaffold layout

Inspired by the 4-layer structure, 2 main strategies were employed in artificial tendon/ligament–bone units: (a) bi-/tri-/quadriphase designs, which construct a discrete stratification of single or several factors [38]; (b) gradient designs achieved by constructing a continuum with biophysical and/or biochemical cues [39], represented in Fig. 1 and Table 2.

**Table 2.** Representative novel strategies about various layouts of multiphasic engineering in tendon/ligament–bone healing. B, bone; CFC, calcified fibrocartilage; CPS, calcium phosphate silicate; HAp, hydroxyapatite; L, ligament; T, tendon; T/L, tendon/ligament; UFC, uncalcified fibrocartilage.

Layouts	Detailed design	Strategies	References
Biphase	Tendon–bone	B phase: bioprints using bioinks containing BMSCs	[148]
		T phase: bioprints using bioinks containing TSPCs (illustrated in Fig. 5A)	
	Bone–ligament–bone	B phase: PLA/deferoxamine@mesoporous hydroxyapatite scaffolds	[226]
		L phase: silk fibroin /connective tissue growth factor@PLCL nanofiber yarn braided scaffolds	
Triphase	Tendon/ligament–cartilage–bone	B phase: calcified collagen type I scaffolds	[95]
		C phase: collagen type II scaffolds functionalized with BMP-2 and TGF-β3	
		T/L phase: collagen type I scaffolds functionalized with PDGF-BB and TGF-β3 (illustrated in Fig. 3B)	
	Tendon/ligament–transition–bone	B phase: melt electrospinning written scaffolds with grid patterns	[92]
		Middle phase: overlap of B phase and T/L phase	
		T/L phase: melt electrospinning written scaffolds with crimped patterns (illustrated in Fig. 3A)	
Quadriphase	Tendon/ligament–calcified fibrocartilage–uncalcified fibrocartilage–bone	B phase: tape casted composite film with a 3:7 PCL:CPS ratio	[113]
		CFB phase: tape casted composite film with a 5:5 PCL:CPS ratio	
		UCFB phase: tape casted composite film with a 7:3 PCL:CPS ratio	
		T/L phase: tape casted composite film with PCL	
Gradient	-	Stiffness gradient scaffold constructed with phototunable polymers by a combination of chemical crosslinking, photocrosslinking, and heat curing	[189]

One biggest challenge for bi-/tri-/quadriphase designs is that potential mechanical instability at interlamination adhesion poses a latent risk during long-term in vivo testing [40]. Conventional preparation involves multiple 3-dimensional (3D) printing or electrospinning to form different layers and finally cross-linking them into a whole. However, these processes fail to guarantee that interlaminar cross-sectional bond is reliable. The macroscopic load will affect the interlaminar microscopic force, resulting in the displacement of each layer, which in turn interferes with the rational force distribution and cell proliferation. In addition, premature collapse does not meet the basic requirements for T/LBJ regeneration. Gradient design can circumvent these problems and provide better mechanical integration. Also, gradient scaffolds hold the most promise for seamless integration [41,42], and therefore are most valued in multiphase engineering.

#### Biphase

Biphasic scaffolds are typically based on 2-zone layout to achieve precise spatial localization of osteogenesis promotion and tenogenesis. The term “biphase” can be interpreted as tendon-to-bone [43–45], or bone–ligament–bone (BLB) [46–49]. The former fits extensive applications, including RC repair, ACL reconstruction, and Achilles tendon healing, while the latter is modeled after ACL, scapholunate interosseous ligament, and patella–patellar tendon complex. However, absence of fibrocartilage layer incurs structural defects compared with natural tissues. It is difficult for tendons/ligaments to connect smoothly with the bone part, and precise regeneration of fibrocartilage is rarely observed.

#### Triphase and quadriphase

In comparison with 2-phase scaffolds, triphasic layouts supplement transition zones or cartilage phase [50–54]. The former involves overlapping the dual phase as a cushioned area, thereby enhancing load transfer. This progressive connection prevents misalignment between tendons and bones, while moderate compliance facilitates the dispersion of shear forces along the coordinate axis, alleviating stress concentration. Accurately reproducing the bridging role of fibrocartilage is considerably more challenging. Recent establishment of this phase universally incorporates supplementation of ECM components, chondrogenic nutrients, and architectural simulation.

The quadriphase engineering pursues comprehensive replication of the 4-layer difference. Moderate mineralization and addition of chondroitin sulfate (CS) and hyaluronic acid prevailed in middle phase construction [55–57]. While the 4-phase layout mostly narrowed the gap between discreteness and gradient, fundamental problem of mechanical instability remained unsettled. Furthermore, cumbersome preparation procedures impede both mass production and clinical translation.

#### Gradient

As an advanced multiphase layout, gradient design can be regarded as a collection of countless smoothly transitioning phases. Macroscopically, there is no apparent stratification, thus preserving the characteristics of the natural microenvironment [58]. Compared to abrupt interlamination, scaffolds with smooth transitions exhibit superior biomimetic properties and promote effective regeneration [59]. Ameliorated mechanical distribution addresses the issue of poor interphase integration by avoiding excessive adhesion and implant slippage caused by insufficient binding. Smooth transitions are facilitated in compression, shear, and tensile properties both before and after enthesis [60]. Considering these aspects, gradient scaffolds find wide application in animal models with well-verified therapeutic efficacy; hence, they provide the most promising prospects for clinical researches.

## Biophysical and Biochemical Cues

The primary objective of tendon/ligament–bone engineering is to physiologically restore interfacial tissue. These artificially assisted instruction can be categorized into 4 main aspects: topography, composition, mechanical properties, and cargoes (Fig. 2C). While previous studies have predominantly focused on individual characterizations, composite modifications have emerged as a promising current trend.

### Multiphasic topography

Biomaterial topology emphasizes the combination of geometric advantages and intrinsic material properties to enhance bioaffinity and tissue regeneration promotion [61]. For 3D structures with specific morphologies, biomaterial topology can act as excellent “mentors” in stem cell fate determination.

#### Fibers

##### Fiber orientation

The arrangement of fibers has been shown to produce distinct signals at the tendon/ligament-to-bone interface. Through the directional assembly of anisotropic fibers obtained by electrospinning, the scaffold was designed to mimic the viscoelastic properties of tendon tissue, resulting in increased graft stiffness and strength. In addition to the improved mechanical properties, the use of topographic cues to guide cell differentiation has also attracted widespread attention [62,63]. Aligned construction positively influenced the tenogenesis of mesenchymal stem cells (MSCs) and TSPCs. In contrast, enhanced osteogenesis and chondrogenesis were closely associated with random orientation, leading to higher levels of alkaline phosphatase (ALP) activity [64,65]. Responses such as the up-regulation of tendon-specific gene expression and the synthesis of Col I indicated a commitment to tendon development on the uniaxially arranged fibers [66]. Conversely, it has been demonstrated that randomly oriented fibers influence FB morphology and trigger degenerative ECM remodeling.

Diverse biomimetic structures were established by combining aligned (A) and random fibers (R). The A–R layout, recommended by early researchers [43,67,68], and the R–A–R layout, resembling BLB composite [46,69], were extensively used in ACL reconstruction models. Partially sonicated fibers showed a distinction between arranged and staggered phases under cross-sectional scanning electron microscope (SEM). Anisotropic and isotropic fabrics simulated the morphological differences between tendon and bone [70]. However, disorganized fibers had lower Young’s modulus and ultimate tensile strength, which resulted in abrupt interlamination changes. To avoid region-wise mechanical concentration, a transition region (T) was introduced as buffering configurations to form an R–T–A–T–R pattern [71,72]. However, fibers in the transition region practically arranged in a circular direction contributed to fabricating deficiency [72]. The concept of an orientation-varying continuum was initially introduced in 2012 [73]. By employing mobile deposition, an R–A gradient was created on the scaffold surface to enhance the potential for heterogeneous cell growth. Two independent teams subsequently reported gradient scaffolds with decreasing changes in fiber diameter, at a scale of 100 nm [74,75]. Particular emphasis was placed on achieving smooth transitions in fiber deviation angles. Even after hydration, the gradient morphology exhibited exceptional durability [76].

##### Microstructure

Further excavation of the tendon/ligament–bone microstructure inspires more biomimetic and sophisticated designs, including wavy artificial ligaments and mesh bone substitutes. Investigation into ACLs revealed that extracellular fibers originally exhibit random orientation but subsequently align from the insertion site and unfold in a wavy pattern within the central ligament to prevent axial deformation [77–79]. Tendons and ligaments possess crimped topography to cushion against increasing tensile strain and enable spring-like movement [80–83]. The unique “toe region” observed in the initial stress–strain curve when stretching the ACL indicates that its curl architecture decentralizes applied load [78]. In vivo experiments confirmed that crimped structure fueled tendon regeneration while inhibiting fat infiltration and inflammation [84,85].

By combining electrospinning and thermal shrinkage, the stress–strain properties and Young’s modulus of wavy fibers were tested, which resembled in vivo ACLs [78]. Wavy-aligned-random fiber-oriented triphasic scaffolds were organized into a cylindrical shape and implanted in rabbits after ACL removal. Col II and Col X deposition as well as tendon regulatory protein gene up-expression were confirmed in the wavy region, while only Col I was enriched in the aligned region. In stem cell and ligamentocyte co-colonization, substrates were divided into axial and wavy regions, resulting in cell behavior similar to natural ACLs [86]. Fibers were printed into discontinuous geometry from horizontal arrangement on one side to sinusoidal amplitude shape on the other [87]. Spatially graded fibers exhibited segmental changes in strain and elasticity modulus.

**Fig. 3.**
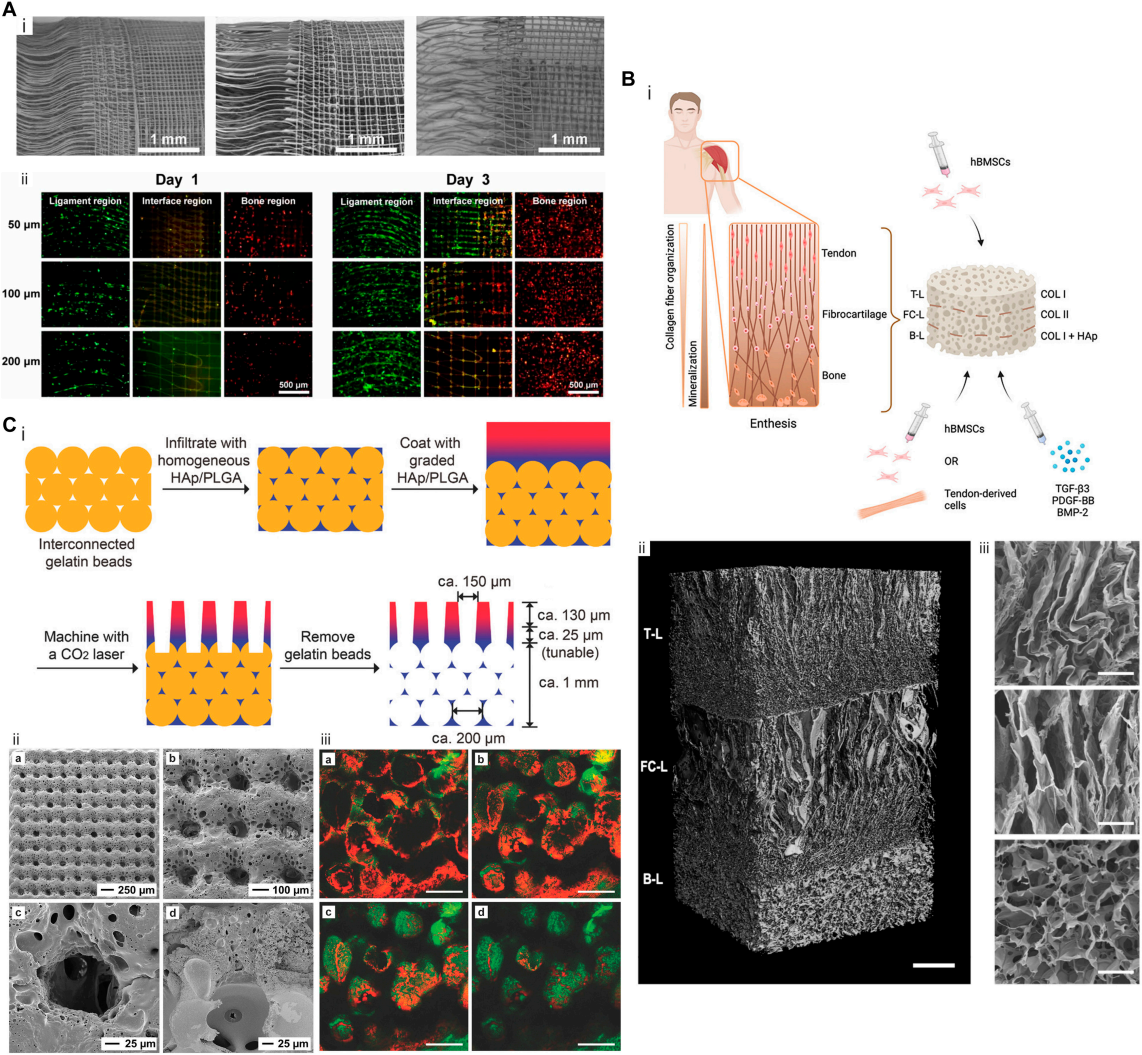
Schematic diagrams of osteotendinous multiphasic scaffolds with specific topography. (A) (i) SEM analysis conducted on crimped-to-grid fabrics with various fiber spacings. (ii) Images of fluorescently labeled NIH/3T3 cells (green) and Saos-2 cells (red) on days 1 and 3 of the curly micropattern (ligament region), mesh micropattern (bone region), and interface region. Reproduced with permission [92]. Copyright 2022, IOP Publishing. (B) (i) Graphical abstract of 3-layered collagen scaffold. (ii) Micro-computed tomography (CT) (scale bar, 1 mm) and (iii) SEM images (scale bar, 50 μm) indicating the longitudinal cross-section of trilayered scaffold. Hierarchies include morphology (slender to round) and pore distribution (loose to dense). T-L, tendon layer; FC-L, fibrocartilage layer; B-L, bone layer. Reproduced with permission [95]. Copyright 2023, Elsevier. (C) (i) Scheme of constructing tunnel-to-sponge gradient scaffold. (ii) (a and b) SEM images of the graded scaffold after template removal. (c and d) A magnified and cross-sectional image showing the morphology of a single channel and the interconnectivity among 3 phases. (iii) Scx (red) and Runx2 (green) fluorescent staining of adipose-derived stem cells seeded after 28 d of incubation in (a) unmineralized zones, (b) transition zones with less mineral content, (c) transition zones with more mineral content, and (d) highly mineralized zones. Reproduced with permission [100]. Copyright 2018, Wiley.

Grid micropatterns resemble porous structures and exhibit similar effects. Several studies have demonstrated that mesh networks are beneficial for osseointegration [88,89]. Utilizing fused deposition modeling, the nozzle’s extrusion track was intricately interwoven to directly construct a grid structure on the end of the parallel fibers [90]. By integrating 3D printed mesh squares with soft electrospun filament, biphasic scaffolds were effectively interconnected in vivo dissimilar tissues [91]. Grid-like scaffolds with appropriate fiber spacing promoted highly expressed osteogenic phenotype markers in Saos-2 cells [92]. Furthermore, these researchers demonstrated a positive correlation between FB orientation angle (inferred from nuclear orientation) and fiber curl angle. Through alternately printing grid and crimped micropatterns (Fig. 3A), a triphasic scaffold was fabricated. Migration of osteoblasts and FBs toward the central region was observed, although they had been seeded at different ends.

While fiber alignment has been the focus, the significant role of crimped fibers in the ligament has been emphasized in recent years, and there have been notable achievements in in vivo studies. However, there is an urgent need for optimal parameters for wavy structures, especially when collapse is observed in the curled section [87]. At the same time, there is a lack of coordinated analysis of the various topological factors. Furthermore, the stress concentration phenomenon is worth noting during ultimate tensile testing, due to the regional mechanical variance. In summary, despite the relatively long history of research, the potential of fiber topology remains quite extensive.

#### Pore

In addition to fibers, biomaterial topology highlights the multidimensional effects of scaffold porosity. A bionic scaffold with appropriate porosity serves as a replacement for the cytoskeleton in the initial phases of bone healing. In comparison to a low-porosity environment, cells cultured in porous scaffolds exhibited an elongated spindle-like morphology and more efficient migration [93]. In another aspect, tendon repair also requires specific 3D geometry to guide the elongated deposition of collagen. Increasing the depth of the pore-like structure can create more space for cell growth. The appropriately organized tunnel structure facilitates efficient adhesion of tendon cells, resulting in a higher specific surface area.

By precisely controlling the exposure time to ethanol vapor in fiber swelling modulation, a novel 2D construct of electrospun poly(lactic-co-glycolic acid) (PLGA) with controllably graded porosity was reported [94]. The transition from a porous nonwoven mat to a dense film was observed. This innovative steam-induced welding technique represents a new approach for fabricating gradient pore-rich scaffolds. Similarly, in a 3-layer scaffold prepared by freeze-drying, the nonuniformity of HAp made the bone layer appear as a compact structure with closely spaced cells [95]. In contrast, cells in fibrocartilage and tendon layers were sparsely arranged and well oriented (Fig. 3B).

The pore parameters were adjusted to mimic the unique characteristics of tendon/ligament–bone insertion. Taking into account the topographic guidance recommended by previous studies, the optimal pore sizes for promoting osteogenesis, chondrogenesis, and tenogenesis were determined as 300 to 400 μm, 150 to 250 μm, and 150 μm, respectively. Eventually, the pore diameter of the bone phase was recommended for 300 μm, twice as large as that of the tendon phase [96]. By setting stitch-related parameters, the embroidery demonstrated triple porosity and pore size, decreasing from the bone zone (average pore diameter: 298.8 μm) to the ligament zone (average pore diameter: 155.7 μm) [86]. In a bilayer design applied for FB and osteoblast coculture [97], the pore size of the bone affinity layer ranged from 90 to 110 μm, while that of the pro-tenogenesis layer was downsized to 5 to 40 μm. Pore density and size were also tunable according to the number and scale of porogen.

In contrast to the linear arrangement of the fiber matrix, the pore structure possesses spatial advantages. First, pore orientation ranked among the elite candidates for fiber secretion and integration guidance. Anisotropy was observed on the tendon side, while disordered orientation was found on the bone side in porous multicompartment bioreactor [44,98,99]. In another creative design, Zhu et al. [100] filled the opal lattice with gelatin beads, and then used HAp/PLGA composite materials to penetrate through the gaps in the lattice to initially prepare a scaffold with outstanding rigidity. To create uniaxial channels at the tendon end of the scaffold, laser technology was employed by the researchers, generating an array with an average pore size of 127 μm. This well-ordered arrangement closely resembled native tendon fascicles and facilitated organized deposition of collagen fibers as well as guided penetration of tendon cells. At the bone end, gelatin beads measuring approximately 220 μm in diameter were dissolved in warm water, leading to the formation of numerous spherical voids with similar dimensions that effectively mimicked trabecular bone’s porous structure and promoted osseointegration. Consequently, a tunnel-to-sponge hierarchy (Fig. 3C) was established to emulate efficient connections between tendon and bone.

Whether spatially graded construction can provide mechanical stability is also a hovering challenge, especially in the bone area with high pore rate requirements. In a gradient porous design, the porosity, pore size, and specific surface area of the material were altered along with the volume of the aqueous dispersed phase in the emulsion, but researchers emphasized the good tensile properties especially at the interface [101]. In view of complication in fabrication and inability for mass production in current researches, more convenient methods for adjusting the internal geometry are expected in the future.

### Multiphasic components

Biochemistry encourages to replicate cell growth environments by applying specific chemicals onto scaffolds. Early researches mainly focused on “addition”, which involves the artificial reproduction of the inorganic (minerals) and organic (collagen, glycosaminoglycan, etc.) microenvironment of the osteotendinous ECM. In recent years, the “subtraction” strategy, which involves removing harmful substances from natural tissues while retaining the active ingredients, has garnered significant attention due to advancements in bioengineering technology.

#### Mineralization

##### Hydroxyapatite

HAp comprises the vast majority of the mineral constituents in human bones but faces a sharp reduction in tendon. Mineralization gradient allows reasonable dispersal of biological forces and directs cell migration and differentiation to spatially deliver cellular phenotypes. Therefore, imparting embedded mineral cues for scaffolds stays evergreen.

**Fig. 4.**
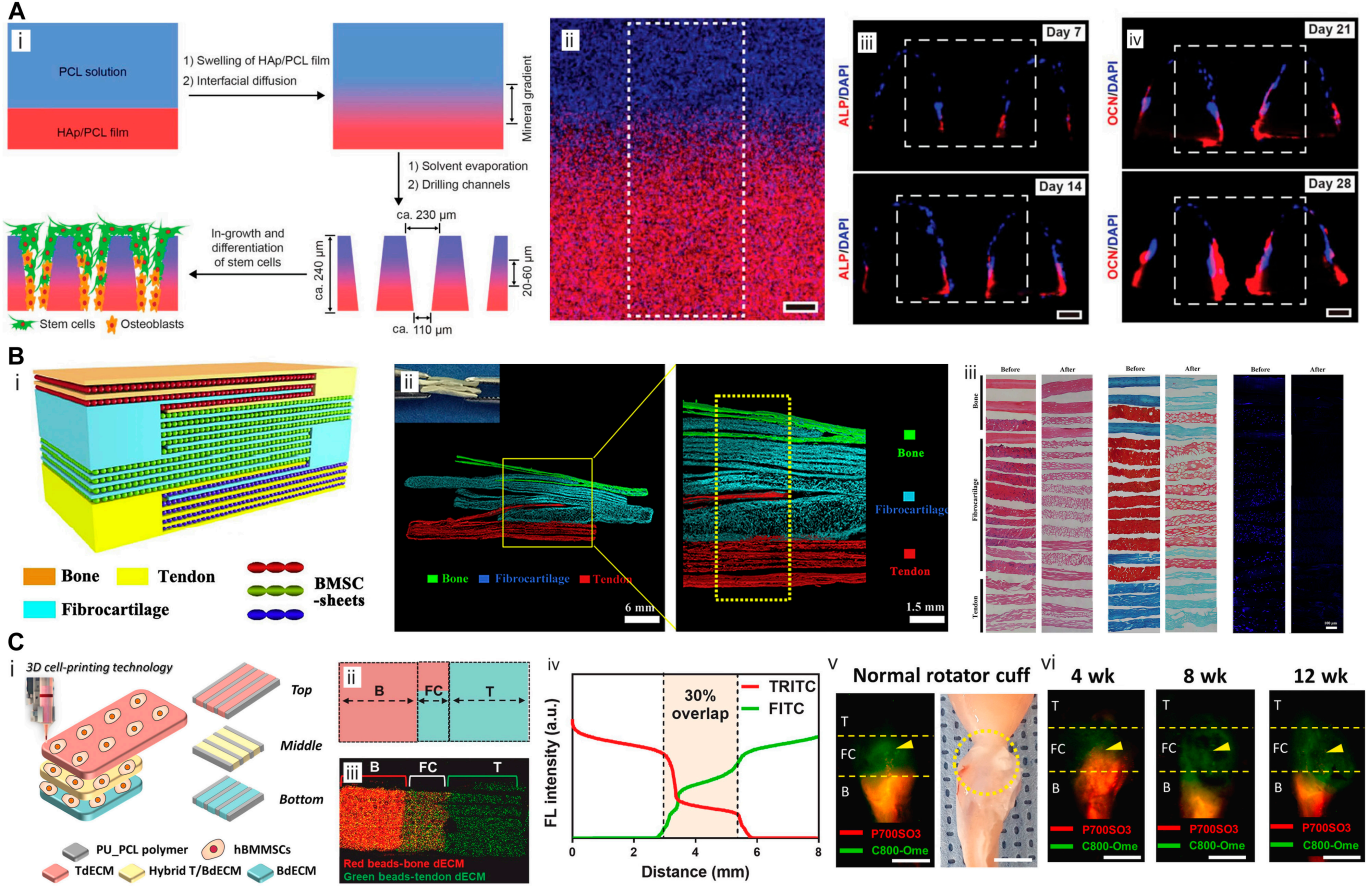
Schematic illustrations of representative biochemical cues provided by multiphasic engineering in T/LBJ reconstruction. (A) (i) Scheme of mineralization gradient establishment by suspension layering and gravity-mediated molecular diffusion and (ii) merged EDS mapping indicating carbon (blue) and calcium (red) distribution. (iii) Cross-sectional fluorescence images of ASCs in scaffolds after osteogenic differentiation at 7 and 14 d. ALP and nuclei were stained red and blue fluorescence, respectively. (iv) Cross-sectional fluorescence images of ASCs in scaffolds 21 and 28 d after osteogenesis. Osteocalcin and nuclei are stained red and blue, respectively. Reproduced with permission [103]. Copyright 2022, Wiley. (B) (i) Schematic illustration, (ii) cross-sectional SR-μCT, (iii) H&E, (iv) Safranin-O–Fast Green, and (v) 4′,6-diamidino-2-phenylindole (DAPI) staining images of bone–fibrocartilage–tendon dECM complex, demonstrating triphasic book composite. Scale bar, 100 μm. Reproduced with permission [143]. Copyright 2020, Elsevier. (C) (i) Scheme of trilayered bioprinting using tendon and bone bioinks and their hybrid. (ii) Layout, (iii) fluorescent mapping, and (iv) linear intensity profile in a top-down view. (v) Dual-channel NIR images of the RC in normal rats and (vi) repaired RCs in the graded scaffold groups at 4, 8, and 12 weeks postoperatively. Images were taken 8 h after C800–OMe (green) and P700–SO3 (red) injections. Yellow arrows indicate the formation of new fibrocartilage between the tendon and bone. Scale bar, 5 mm. Reproduced with permission [147]. Copyright 2022, Elsevier.

Graded mineralization was a classic concern of multiphase production. Building upon this concept, yarns with varying HAp content (0%, 5%, 10%, and 15%) were electrospun to construct scaffolds with 4 distinct zones [55], which was macroscopically reflected in a gradual roughness increase. Spatially controllable presentation of HAp on woven products determined tri-lineage fate of bone marrow stem cells (BMSCs). Equivalent effect can be detected in narrower HAp concentration gaps (0% to 2%) in hydrogel scaffolds [57]. Naturally, enthesis forms continuous mineral deposits over a length of 20 to 60 μm [102], which necessitates optimal reproduction of this gradient. Molecular diffusion phenomenon was ingeniously utilized to fabricate scaffolds with gradient HAp content (Fig. 4A) [103]. HAp and poly-ε-caprolactone (PCL) were primarily solved at a 1:1 mass ratio to form a membranous structure. This was followed by second PCL diffusion on top of the film. Similarly, the heated HAp solution was suspended on a porous lattice [104], progressively penetrating under the support of gravity, which was captured by the subsequently poured PLGA solution. The overheating process solidified the lattice structure, which influenced nanoparticle penetration depth, thereby enabling vertical control over gradient formation.

Deficiency in degradability is a limitation of mineral gradient scaffolds. Existing methods include doping HAp in degradable materials like PLGA. Two PLGA/PCL inks [105], one containing HAp and the other HAp-free, were 3D-printed at both ends of the scaffold frame, maintaining good flexibility. Moreover, implants after reconstructive surgery revealed that approximately 30% of the scaffolds had degraded at 6 weeks, increasing to 60% at 12 weeks. Given the remarkable repair effect, future research needs to balance the degradation rate and mechanical properties. For example, the optimal ratio of HAp to substrate materials may vary in different areas of the scaffold.

##### Other CaPs

Among the interesting materials for hard tissue engineering, CaP, such as β-tricalcium phosphate, and CaP salts structurally resemble natural bone [106]. As a representative of the in vitro mineralization process, immersion in simulated body fluid (SBF) can functionalize material surface to mimic human physiology. Controllable precipitation allows for gradient mineral deposition, which plays a role of pioneer in the early researches of T/LBJ gradient scaffolds [107–111]. An interesting strategy for the transformation of soft tissues to hard tissues by biomimetic mineralization has been proposed [112]. The tendon tissue is soaked in an amorphous CaP (HAp precursor) system before being transplanted. Unlike the disordered calcification process in the above studies, the mineral particles penetrate the collagen and nucleate internally, which significantly improves the mechanical properties and osteoinducible ability of collagen fibers and forms a jagged interface in vivo.

In addition to liquid immersion, there are alternative cosynthesis methods for in vitro mineralization. Tape casting is extensively used in the production of functional gradient bioceramics because it allows for the orderly incorporation of inorganic powders and organic substrates through adjustable parameters. In the study of Su et al. [113], the gradient of calcium phosphate silicate (CPS) was customized in multilayered PCL fibers at different CPS/PCL ratios (0, 3:7, 1:1, 7:3). Integration between compartments containing CaP and CaP-free materials was achieved through suspension diffusion [68,99,114]. By combining hybrid twin-screw extrusion with electrospinning, the intermittent bonding of tricalcium phosphate (TCP) nanoparticles to PCL was achieved [115]. In layer-by-layer printing, increasing the ink extrusion speed enabled the fabrication of a 3-layer scaffold with varying TCP content [116]. More intriguingly, employing reverse thinking, gradient demineralization can also serve the purpose of gradient mineralization [117–119]. The researchers immersed a portion of the sections of the acellular matrix in a decalcification agent (such as hydrochloric acid and EDTA) and then reconnected them with the normal decellularized matrices as necessary.

##### Bioglass

In addition to bone conductivity, bioglass (BG) is known for its dynamic response. BG tends to chemically bond with surrounding bone, or biodegrades to form new bone components, which resulted in its utilizations in bone reconstruction [120]. In multiphasic scaffolds, BG was replenished to the bone phase as the equivalent of HAp or CaP particles [54], or 3D-printed for gradient reproduction [121].

The new generation of products, such as mesoporous bioglass (MBG), demonstrates controllable formation with moderate pore dimensions [122,123], promoting osteoblast proliferation and differentiation, anti-inflammatory effects, and angiogenesis in connective tissue. In layer-by-layer electrospinning [124], different proportions of MBG (0%, 0%, 0.25%, 0.5%, 1.0%, 2.0%, and 4.0%) were incorporated into distinct regions of a 7-layer patch to create a mineral gradient along the vertical direction. Low-temperature nitrogen adsorption–desorption test revealed its higher pore density, specific surface area, and pore size of around 50 nm, which matched the dimensions of tissue-resident cells and fueled their migration and development. Compared to CaP and its isomers, MBG’s excellent hydrophilicity also conferred lower expansion coefficient and higher degradation efficiency. The maintenance of strong interlaminar bonding after 6 weeks suggested potential for achieving a balance between new bone formation rate and long-term structural stability.

#### Other ECM substances

In enthesis, the major collagen fibers start from Col I (tendons) to Col II (fibrocartilage) fibers, and end at calcified Col I (bone) [15,125]. Re-establishing regional collagen distribution initiates the correct process of tendon bone healing. The co-electrospun nanofiber scaffold was separated into the Col I-modified PLGA and PCL with HAp nanoparticles [45]. To promote cartilage growth, Col II or transforming growth factor-β (TGF-β) is integrated into the middle of the Col I layer and the HAp layer to restore natural continuum [126,127]. Given that bone ECM also contains considerable Col I, a more ideal 3-layer structure would follow a Col I–Col II-calcified Col I sequence [95]. The BMSCs exhibited lineage shift potential corresponding to collagen species.

It should be emphasized that few studies have specifically replicated fibrocartilage in the enthesis transition. Although the primary criterion for subdivision is the degree of mineralization, there are also variations in the content of macromolecular inorganic compounds such as collagen, hyaluronic acid, and CS [118]. Amelioration involves the incorporation of HAp nanoparticles in one region [128], and CS and hyaluronic acid in the other 2 phase of the scaffolds. The EDX analysis revealed specific, high concentrations of calcium (in the bone phase) and large deposits of sodium and sulfur. Olvera et al. [53] cotreated tendon and articular cartilage ECM with a PCL triphasic scaffold. Amine-containing molecules, primarily Col I and Col II, were captured by the functionalized surface, and then the bone zone was coated with apatite. Furthermore, replicas of calcified and noncalcified fibrocartilage were produced in poly-l-lactic acid (PLLA) bilayer fibrous membranes [129]. In a study by Kim et al. [56], mineralized fibrocartilage components were differentiated from nonmineralized components by adjusting the ratio of CS and HAp in collagen-based scaffolds.

A comprehensive ingredient gradient was developed in 2022 [130]. The substrate was alternately immersed in positively charged collagen and negatively charged CS solution after oxygen plasma treatment to initiate self-assembly. Since charge neutralization was related to the immersion depth, by adjusting the submerged area, a stepped distribution of proteins and polysaccharides arranged along the linear fibers was produced. BMSCs' preferences toward high content region were observed, demonstrating potential for interfacial tissue application.

#### Decellularized ECM

In recent years, there has been extensive attention on the structural optimization of decellularized ECM (dECM), including its application in tendon injuries [131,132], rupture of the myotendinous junction [133], and fibrocartilage regeneration in tendon/ligament–bone healing [134,135]. However, dECM scaffolds derived from a single tissue source mainly focus on individual tissues within complex structures and are incapable of comprehensively simulating the T/LBJ.

The direct application of dECM multiphases is to assemble different ECMs for stem cell carriage, exemplified by bone–fibrocartilage–tendon design [50,136,137]. Staining and quantitative analysis confirmed that each dECM closely resembled the characteristics of normal tissues. In another approach [138], a porous and interconnected meniscus dECM was combined with demineralized bone dECM to enhance physical properties. Additional modifications include physical (ultrasonication to arrange fibers) [46], chemical (mineralized) [53,139,140], or biological (ECM predeposition or mixing with platelet-rich plasma) [117,141] enhancement to improve graded healing. However, direct implantation did not fully exploit these advantages. When comparing the efficacy between monophasic fibrocartilage-derived grafts and allogenic patellar ligament–patellar complex in chronic RC injury [142], significant differences were only observed during the early postoperative period.

Another effective physical treatment involves slicing the substrate and assembling it to resemble a “book”. The main challenge addressed by this treatment is the dense organization of fibrocartilage and tendon, in contrast to the highly porous bone, which hampers cell migration toward the center. Unsuccessful infiltration also hinders the function of bioactive factors, leading to inadequate regeneration. On the contrary, book-shaped dECM scaffolds offer several advantages: (a) increased surface area, which facilitates the penetration of decellular chemical reagents, thereby reducing exposure time and tissue loss [135,143]; (b) biocompatibility to sandwich seed cells between the pages to form a cell sheet, which is conducive for cell–substrate contact; (c) geographical depth, which contributes to cell adhesion, migration, and accelerated tissue healing; (d) enhanced bioabsorbability, promoting interfacial integration [143].

Currently, there are 2 main strategies for “book” fabrication. The first method is sampling from the tendon/ligament–bone insertion and slicing at the interface. For example, Chen et al. [144] removed the porcine infraspinatus tendon and its attached bone. After aseptic cleaning and decalcification, the junction zone of the enthesis was vertically cut into a book-shaped structure with a layer thickness of 250 μm and a cross-sectional area of 8 mm^2^. Decellularization was then carried out using a customized vacuum aspiration device. Staining experiments, combined with synchrotron radiation FTIR (SR-FTIR) imaging, revealed a regional characteristic distribution of collagen and proteoglycan content similar to that of normal tissue. Likewise, the second method involves using ECMs obtained from bone, fibrocartilage, and tendons. Subsequently, decellularization and re-assembly are performed. Using the spaces between “pages”, “books” were woven together and arranged in sequence to create a gradient scaffold. Tang et al. [143] obtained sections of 3 tissues and sliced them into book-like scaffolds with a thickness of 100 μm per “page” and a “spine” width of 2 mm. The internal structures consisted of 3 “pages” for bone and 5 “pages” for fibrocartilage and tendon. By intertwining “pages”, a phase contrast emerged from the top (1 bone “book”), through the waist (2 fibrocartilage “books”), to the bottom (1 tendon “book”) (Fig. 4B).

dECM-based bioinks are increasingly prevalent in 3D bioprinting due to their solubility. Rheological experiments demonstrated the appropriate shear viscosity of the bioinks separated from tendons and ligaments, sufficient for additive manufacturing [145]. The direct approach involves extracting dECMs from domestic animals to form nongel solutions, enabling scale-controlled bioprinting [96]. Interestingly, Chae et al. [146] prepared only 2 types of bioinks derived from porcine Achilles tendon and bone, and utilized the mixture for the transition region. In a previous study, dECMs of tendon and bone were printed inhomogeneously on polyurethane (PU)/PCL bases, forming gradient stratification in the opposite direction. Two years later, visualization of gradient formation was achieved through excitation of specific fluorescence [147]. For the first time, dual-channel near-infrared fluorescence (NIR) enabled real-time assessment of bone and fibrocartilage during the in vivo RC healing process without interference (Fig. 4C).

Current cell-removing methods primarily focus on physical and chemical aspects; however, the former often lacks efficiency for a thorough removal, while the latter, involving the use of reagents such as Triton X-100 and sodium dodecyl sulfate, leads to inevitable matrix loss that compromises mimicry fidelity. Despite remaining unsolved questions, it is undeniable that dECM construction represents a significant endeavor toward restoring in vivo microenvironment with promising potential.

### Multiphasic cargoes

Ordinarily, in the efficient transportation of bioactive substances, scaffolds act as a “wagon” to deliver the “cargo” in place. Scaffold functionalization must consider preserving biological activity and structural stability while accommodating the specific properties of the tendon-to-bone hierarchy. Delivery systems based on cells, growth factors (GFs), metal ions, and genes have been developed and are described below.

#### Multicellular implantation

One of the main difficulties in in vivo tendon-to-bone healing is hypocellularity. Viable solutions include lineage direction interference of stem cells or transport of exogenous cells. Optional seed cells consist of BMSCs, TSPCs, adipose-derived stem cells (ADSCs), and human umbilical cord-derived mesenchymal stem cells (hUCMSCs).

**Fig. 5.**
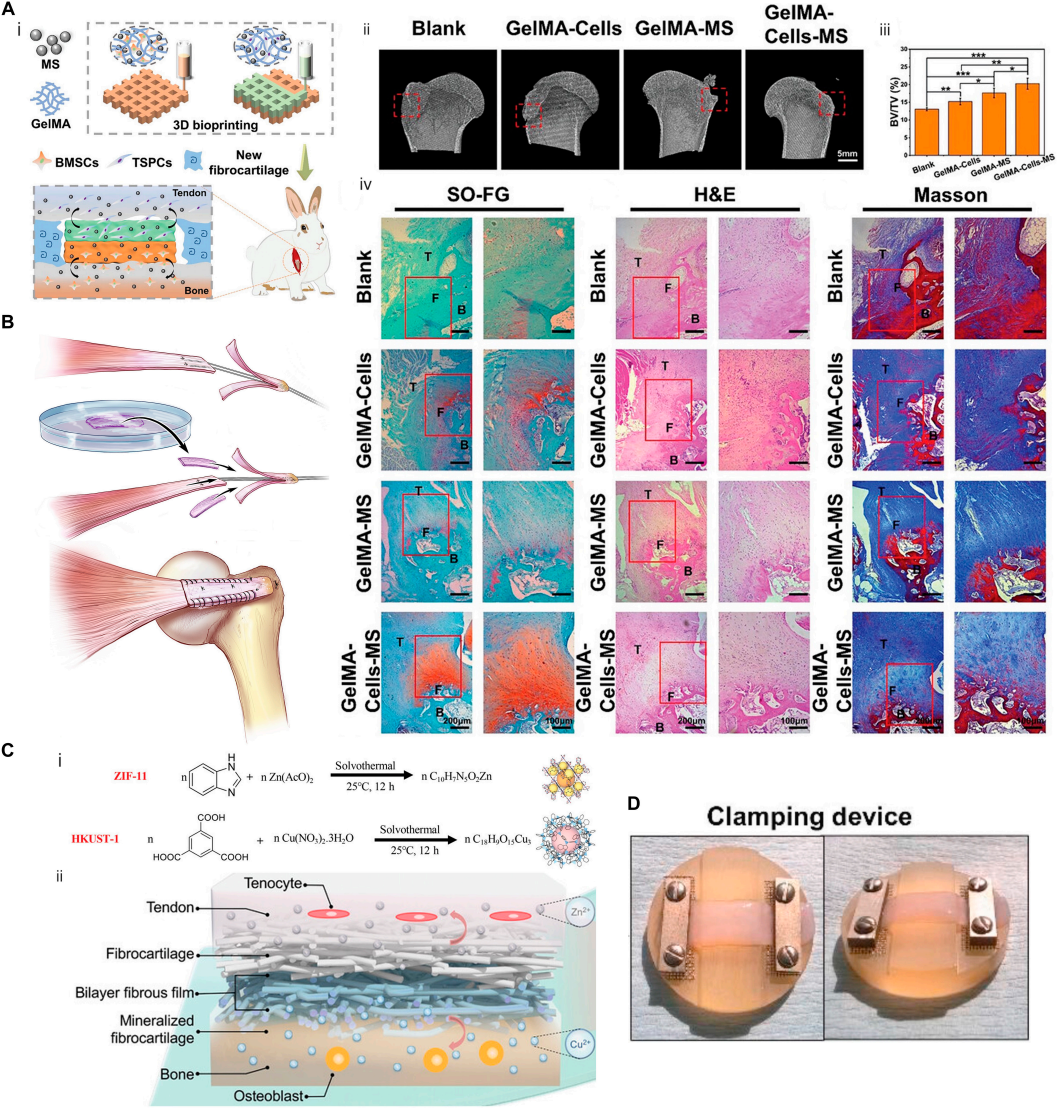
Multiphasic scaffolds that provide additional cues for enthesis repair. (A) (i) Scheme of dual-layered scaffold containing BMSCs (top), TSPCs (bottom), and silicate bioceramics (both). (ii) Representative micro-CT image of the humeral head at 12 weeks postoperatively. (iii) Bone volume fraction values for different groups at 12 weeks. (iv) Representative Safranin-O–Fast Green, H&E, and Masson staining images of regenerated tendon (T), fibrocartilage (F), and bone (B) 12 weeks postoperatively. Reproduced with permission [148]. Copyright 2023, Wiley. (B) Cell sheet sandwiched by tendon–fibrocartilage–bone decellularized matrix to bridge the osteotendinous stumps. Reproduced with permission [50]. Copyright 2019, Elsevier. (C) Schematic diagram of ZIF-11 and HKUST-1 formation and simultaneous promotion for tendon/ligament–bone integration. Reproduced with permission [181]. Copyright 2022, Wiley. (D) Images (top) of compression–tensile–compression mechanical environment by clamping at both ends of cell-seeded collagen. Comparison (bottom) of tissues clamped for 6 weeks or derived from juvenile bulls by atomic force microscopy and low magnification confocal reflectance 3D reconstructions. Reproduced with permission [191]. Copyright 2021, Elsevier.

In multicellular engineering, a classic combination is BMSCs with osteogenic and chondrogenic potential, as well as TSPCs that are more inclined to tendon differentiation. For example, a bicellular scaffold was bioprinted with gelatin methacryloyl (GelMA) bioinks containing either BMSCs or TSPCs (Fig. 5A) [148]. The porous scaffold suitable for cell adhesion consisted of a tendon phase at the top and a bone phase at the bottom, and facilitated repair in RC injuries in rabbits. On this basis, Du et al. [149] integrated manganese silicate nanoparticles into BMSC/TSPC biphasic scaffolds to participate in microenvironment remodeling. Immunomodulatory manganese ions stimulate macrophages to secrete prostaglandin E2 (PGE2), which further promotes the corresponding specific differentiation of multicellular scaffolds. Microfluidic chips were manufactured to create proportional gradients of culture medium [150], which were followed by loading BMSCs and TSPCs. To ensure precise spatial delivery of different cell types, tissue-specific medium can also assist before implantation. ADSCs were precultured for 2 weeks, and their transition into tendon, cartilage, and bone lineages was observed in vitro [105]. However, in-depth understanding of the graft–host response is needed to prevent the pro-inflammatory effects of immune system on exogenous cells [151].

If a longer preculture is attempted, the cell sheet technique can serve as reference. Traditionally, cells are grown in a medium to fuse into tissues and transferred to the injury site as a monolayer augmentation [152,153]. After resident cells scavenge, BMSCs were cultured on the bone–fibrocartilage–tendon composite. Under the combined action of biomimetic matrix and mechanical pre-adaptation, BMSCs fused into plates after 7 d and showed potential for in vivo patching [154]. Detaching canine infraspinatus tendons, the hierarchical cell-free matrices were sutured to bridge the osteotendinous stumps, and BMSC sheets were placed in direct contact with local tissues (Fig. 5B) [50]. Six weeks postoperatively, fibrocartilage, instead of Sharpey-like fibers, was observed at the graft interface. In situ delivery of stem cells compressed the proliferative space of granulation tissues, thereby delaying the mechanical instability caused by scar formation. Different medium formulations induced osteogenesis and tenogenesis of ADSCs, respectively [137]. Later, the 2 cell sheets were stacked into an in vitro construct of T/LBJ. In another study, cell sheets were generated after 21-d ex vivo culture, which were wrapped as bundles and seeded in a tri-compartment scaffold imitating ligament–bone–ligament junction [48].

#### Bioactive molecules

##### Growth factors

During enthesis development or repair, a multitude of cytokines orchestrate the homeostasis and gradient regeneration under the complex regulation of signaling pathways [40], including (a) bone morphogenic protein (BMP) for bone remodeling [155]; (b) TGF-β for cartilage regeneration [156]; and (c) connective tissue growth factor (CTGF), platelet-derived growth factor (PDGF), fibroblast growth factor (FGF), and growth and differentiation factor (GDF) for tendon healing [157–160]. The advantage of multiphasic scaffolds lies in the micro-precise spatiotemporal delivery of GFs required at tissue junctions, thereby encouraging gradient restoration of cell phenotypes along with desired direction.

Regulation by BMP superfamily members is almost ubiquitous in bone-related structures [161]. BMP-2 content transition [71,74] or reverse gradient with other GFs (PDGF-BB [162], TGF-β1 [163], TGF-β3 [164], etc.) has received continued attention. Other members, BMP-4 and BMP-12 (also known as GDF-7), separately participated to the construction of bone and cartilage phases [165]. Among the candidates for tenogenesis, CTGF is highly favored in multiphase engineering [166–168]. Through 3D printing, BMP-2, TGF-β3, and CTGF were encapsulated in degradable microspheres and embedded with 3-layered PCL microchains. Subregional release was achieved in acute [167] and chronic [166] RC tears, and effectively promoted the insertion of supraspinatus tendon into humerus. The role of FGF in promoting vasculogenesis and tenocyte growth has been well established [169]. Microspheres loaded with BMP-2, TGF-β3, and basic fibroblast growth factor (bFGF) were precisely bioprinted onto distinct regions of the substrate using a microfluidic system along with hUCMSCs [170]. Novel delivery systems, such as ultrasound-controllable cerasomes, loading BMP-2, TGFβ1, and FGF-2 [171], can be precisely released to the site of injury to improve healing.

Material surface modification is one of the most common strategies for constructing multiphase GFs. The catecholamines of polydopamine (PDA) can react with amines and thiols in many biological factors through Schiff base reaction or Michael addition [172]. Thus, gradient PDA modification can realize inhomogeneous PDGF-BB immobilization [108]. Heparin-mediated gradient links were also created with GFs [162,173]. In details, PCL can expose carboxyl groups through surface alkaline hydrolysis, and after activation, formamide reaction occurred to attach different kinds of collagen [53]. Innovated hierarchical synthesis of BMP-2, TGF-β3, and GDF-7 on decellularized matrices was reported by the collagen-binding peptide fusion into the N terminus of GFs [144]. A controlled coating-enhanced FB adhesion by click reactions of thermo-activated thiol-yne, copper-free alkyne and azide, and graded distribution of BMP-2 and FGF-2 in opposite direction was reported [174]. The multizonal-tethered effect and sustained release were validated by a modified enzyme-linked immunosorbent assay (ELISA) assay. Given the potential biotoxicity, long-term tests are needed for reassurance. The presence only on the scaffold surface also hampered bioactivity retention.

##### Metal ions

Metal ions are widely distributed throughout the human body, providing essential trace elements necessary for various physiological activities. Their indispensable role is evident in maintaining cellular homeostasis, including nucleic acid metabolism, hormone and enzyme synthesis, as well as interfacial tissues repair. For instance, magnesium ions (Mg^2+^) exhibit anti-inflammatory and proliferative functions that have been harnessed in tissue engineering approaches [175,176]. Divalent copper ions (Cu^2+^), on the other hand, promote angiogenesis in osteochondral defects by activating the hypoxia-inducible factor-1 (HIF-1) pathway [177]. Additionally, zinc (Zn^2+^) and strontium (Sr^2+^) have also been reported to enhance enthesis repair [178–180].

Metal ions were embedded with porous crystals in metal–organic frameworks (MOFs), presenting a highly stable and ordered 3D structure. Composed of ZIF-11 (carrying Zn^2+^) and HKUST-1 (carrying Cu^2+^), a biphasic scaffold coupled T/LBJ integration and angiogenesis promotion (Fig. 5C) [181]. Two MOF particles were added separately to the electrospinning solution mixture, manufacturing a double-layer membrane, where fiber interconnected yet clearly demarcated in macroscopic view. To ensure the specific effect of metal ions, tendon- and cartilage-targeting peptides were fixed on ZIF-8 and Mg-MOF, respectively, by electrostatic adsorption. Osteogenic differentiation of BMSCs induced by Zn^2+^ and enhanced Col II secretion induced by Mg^2+^ were observed [182].

Gradient hydrogels are appropriate systems as metal ion conveyance. Spatially variated presentation of metal ions was realized with mineral sheets [183]. Laponite, a silicate containing lithium and magnesium, exhibited appropriate swelling ratio and hydrodynamic properties when dissolved in water. Therefore, it was discretely dispersed in a 4-layer photosensitive gel precursor at concentrations ranging from 0 to 2.5%. Increasing levels of Li, Mg, and Si were detected after curing, and inductively coupled plasma analysis indicated the 1-month sustained release. In another study introducing copper and zinc, sulfhydryl groups in thiol gelatin coordination-crosslinked with metal ions [184]. EDS characterized the reverse trend of Zn^2+^ and Cu^2+^ distributions. Apart from self-healing and biodegradability, the scaffold maintained the delayed release of Zn^2+^ and Cu^2+^. Thus, antimicrobial properties were conferred to the scaffold, avoiding post-implantation inflammation and septic loose.

Restriction lies in the lack of demonstrated long-term therapeutic efficacy. Due to the extremely low requirement of metal ions in the body, appropriate dosage is expected to administer for interface tissue engineering, especially refraining from side effects, such as interference with cell metabolism, genetic variation, or even damage to other tissues. The feasibility of clinical translation motivates more large-scale and comprehensive long-term in vivo studies.

### Multiphasic mechanical environment

The exploration of multiphase stiffness gradually sprang up by regionally altering the mixture proportion of raw materials. Scaffolds constructed from electrophoretic gels with varying concentrations have demonstrated gradient compliance, as evidenced by a larger distribution area of BMSCs in regions with high elastic modulus and consistent results in Runx2 expression assays [185]. In a dual-feed crosslinking system, gradient hydrogel matrices with significantly varying moduli of compression (from 12 to 306 kPa) were fabricated by controlled dilution of polyethylene glycol solution [186]. Another stiffness gradient scaffold was produced via 3D-printing by altering the ratio between 2 commercial materials with different shore hardness values [187]. Results indicate that specimens with linear graded stiffness exhibit well-proportioned strain distribution and stress–strain curves that grow linearly in the initial stage, which is critical for maintaining stability.

When assembling parts with different stiffness, stress concentration phenomenon at the interface deserves attention, which can be mitigated through a smooth transition. After graded photocrosslinking treatment of PU network, an increasing stiffness (0.6 to 2.7 GPa) was observed on a scaffold with a span of 1 to 2 mm [188]. The regions with higher stiffness exhibited enhanced ALP activity, while areas with lower stiffness demonstrated more significant expression of tendon cell markers; this effect could be reversed by employing the mechanotransduction inhibitor. In another study on PU network [189], the effective role of stiffness gradients in reducing stress concentrations was highlighted. In finite element analysis and photoelastic tensile testing, a gradual reduction in stress from hard to soft areas was investigated, aiding the anchoring of tendons onto the bone. Reasonable topological design with substrate progressive stiffness changes can also reduce the stress of hard-to-soft microstructures. In order to improve the rivet system commonly used in clinical practice, another batch of PU-based materials was prepared into a screw structure with stiffness rising first and then descending [190]. Compared with the control group with constant stiffness, the finite element analysis showed that the peak stress concentration coefficient was reduced by more than 40%, which was verified in the rabbit RC injury model.

Compared with the direct preparation of multiphase stiffness, the self-made force-applying device may be better suited. In physiological condition, tendons were mostly stretched, while osteochondral tissues need to face compression challenges, and insertion sites resist against rotational shear. A culture system based on aligned collagen gels was developed [191], with both ends clamped to create a compression–tensile–compression mechanical niche (Fig. 5D). After 6 weeks of culture, tenocytes, ligamentocytes, and chondrocytes secreted fibers (average diameters of 22, 30, and 37 μm) and glycosaminoglycan that matched their respective physiological conditions, and the fibril microstructure was further confirmed. The harvested tendon and ligament tissues rendered tensile properties matched to those of juvenile animals (more than 1 MPa), and the toe region was observed in the stress–strain curve. In order to approach the Young's modulus of natural tissues, lysyl oxidase was first supplemented in culture condition to initiate fibril trivalent crosslinking and hierarchical assembly [192], which was expected in functional recovery at the soft–hard interface.

Enthesis is located at dynamic mechanical surroundings. Repeatedly intermittent tensile strain was adopted by tendon engineering, and the mechanisms regulating collagen types and catabolism were verified [193,194]. Circulating force (10% strain for 10 min, 1 Hz, per 6 h) was applied in multiphasic bioreactors to explore its role in fate determination [68]. In the tendon phase, mechanosensitive pathways were monitored, including activation of extracellular signal-regulated kinase (ERK1/2) to enhance collagen deposition and down-regulation of inhibitory p38 mitogen-activated protein kinase (MAPK). In the osteogenic region, Smad 2/3 and Smad 1/5/8 were launched to assist bone regeneration. High expression of chondrogenesis marker Acan and Sox9 was detected at the intersection exposed to cyclic testing. Stem cell morphological analysis showed that static stretching improved the nuclear aspect ratio, nuclear arrangement, and actin arrangement at the anisotropic end, presenting an FB-like shape [195].

Dilemmas of graded mechanical design include that the range of tensile characterizations most conducive to tendon regeneration maintained to be in controversy. Contradictory to the distinct of replicating physical condition, stiffness that is significantly lower than in in vivo tendons was also emphasized. Comparative studies spanning different elastic modulus of magnitude are expected to suggest the ideal mechanical conditions. Furthermore, given the limited distance (0.1 to 1 mm) of T/LBJ, it is necessary to apply microindentation tests in stiffness characterization for more precise determinations [187,188].

### Multidimensional cues

Internal construction at heterogeneous tissue junctions is widely acknowledged sophistication. To meet specific functional needs, the microenvironment of living organisms found biophysical and biochemical gradients in multiple dimensions. In the early researches for tendon/ligament–bone repair, multiphasic scaffolds mainly focused on individual gradient. However, with advancing technology and deeper understanding, cues from various categories have been intentionally integrated into a single unit to provide flexible guidance. Representative novel studies are listed in Table 3.

**Table 3.** Introduction of dual or more dimensional focus in tendon/ligament–bone multiphase engineering

Hierarchies	Cues	Strategies	References
Topography and component	Anisotropic structure and mineralization	Porous structure transitioned to parallel tunnels, which was coated with PLGA solutions with incremental HAp concentrations	[100]
		CaP particles in the upper solution gradually penetrated downward to establish an inorganic content gradient, additionally interfering the pore orientation during directional freeze-drying	[99,195]
	Fiber spacing and mineralization	Graded UV exposure of photosensitive polymers and temporal control of SBF immersion	[258]
Topography and cargo	Anisotropic structure and GFs	TGF-β2 and GDF-5 were carried by regions of aligned and random pore arrangement	[173]
		Recombinant BMP2, TGFβ3, or C-GDF7 is tethered into collagen fibers of decellularized bone, cartilage, and tendons	[259]
Component and cargo	Mineralization and GFs	Gradient mixing of mineral particle and BMP-2 with substrate materials	[68,74,128]
Mechanics and component	Substrate stiffness and mineralization	Scaffolds were prepared by sequential spin coating of nano-HAp/PCL suspension with decreasing concentration, and funnel-shaped microchannel arrays were formed by laser processing	[260]
Mechanics and cargo	Tensile strength and GFs	Light-mediated gradient polymerization and local tensile properties were positively correlated with exposure time, and BMP-2 and FGF-2 were adsorbed at either end of the scaffold	[188]
Others	Anisotropic structure and piezoelectric coefficient	Electrospinning back-to-back layers of PLLA/zinc oxide (tendon phase) and PLLA/barium titanate (bone phase), and manipulating the rotational speed of the rollers to arrange is adjusted from aligned-to-random fibers	[246]
Comprehensive applications	Collagen, Ca content, and ECM protein	Decellularized matrix	[144]
	Pore diameter and substance content	Bioinks from different tissues were printed into porous scaffolds of varying diameters	[96]
	Anisotropic structure, mineralization, and GFs	Dual-channel electrospinning adjusted the emulsion mixture of HAp nanoparticles and BMP-2 content; fiber transition from parallel extension to chaotic distribution was observed	[71,261]
	Anisotropic structure, GFs, and other ECM substances	Electrospun aligned-to-random layered composite nanofiber patches with random layers injected with enzymatic hydrolysates from acellular Wharton jelly tissue	[262]

Notably, the factors of structure, composition, mechanics, and cargoes are intricately interconnected. In multiphase design involving multiple features, different nodes were interwoven into a network and harmonized to present unique characteristics (Fig. 2C). This novel perspective inspired us to reevaluate multiphase scaffolds. First, the interaction between structure and components is evident. The introduction of mineral nanoparticles intensifies pore formation and fiber orientation [124,196]. After lyophilization, anisotropic pores in the unmineralized region were not observed in the mineralized region, indicating the slowed formation of ice crystals in the bone phase [99]. Hypothesis was raised about the increased viscosity of the mineral-containing collagen suspension and the convection-mediated cooling of the upper solution by the freeze-drying chamber.

Second, the above 2 cues form the basis of scaffold mechanics; however, their synergistic effects exhibit some inconsistencies. The mechanical testing of randomly and orderly oriented and crimped fibers indicated a gradual trend of strengthening in tensile property identifications [78]. The signature toe region arose contributing to the curly microstructure of artificial ligaments, where strain occurred easily at the beginning and corresponded to the natural ACL (3 to 4 mm displacement or 10% to 15% estimated strain). Fiber deformation can be adjusted by the corrugation ratio or wavelength. Mineralized gradient scaffolds were prepared with additional biphase fiber distribution and/or tensile strain for orientation examination of MSCs [195]. The results indicated that fiber arrangement effectively regulated the transition of MSCs from spindle-shaped to round morphology, while stretching only significant controlled the anisotropic areas.

Structure and composition more directly affected the regulation of matrix stiffness. Gradient stiffness matrix was detected in mineral content regulation [197]. By analyzing the surface coating of gradient SBF immersion, Lipner et al. proposed that calcium absorption increased material tensile properties [198]. Along the gradient of reduced calcification deposition, a decrease in Young’s modulus from approximately 2 GPa to 300 MPa was observed [104], consistent with a physiological transition from rigid to flexible.

Third, the cargo detachment process is intimately related to the architecture, and in turn affects the mechanical properties of the scaffold. Typically, bioactive substances first experience explosive large-dose surges, followed by sustained release. In view of the short half-life of bioactive substances, and the side effects of ectopic ossification and chronic inflammation caused by excessive release, it is of great significance to strictly control spatiotemporal presentation. Through the opposite charges of chitosan and alginate, different levels of self-assembled coatings were constructed on the surface of PCL membranes [164]. After ELISA and BMP-response fluorescence quantifying GF release, it was found that the BMP-2 release time was gradually elongated with the increase of layer number, and reached the top at 5 layers. For dense pore structure transportation, by controlling pore number and diameter, the release rate of the encapsulated GF microspheres can be tunable for different needs. Adjusting the material ratio helped to achieve orderly internal decomposition of the scaffold [199], but this necessitates meticulous design to equalize stability and mechanical support. In summary, achieving accurate repair through comprehensive utilization of various cues represents the ultimate goal in multidimensional multiphasic engineering.

## Fabrication Strategies

Fabrication processes play a crucial role in tailoring the structural and compositional properties of scaffolds, which is essential for achieving tendon/ligament–bone repair. The current approaches employed in this field can be summarized as follows: (a) techniques for creating biomimetic structures and replicating the T/LBJ structure and components, including electrospinning, 3D printing and bioprinting, and textiling; (b) techniques for stacking multiphasic scaffolds and integrating each layer with hierarchical structures, especially freeze-drying and photo-assisted or chemical crosslinking. This section provides an overview of main fabrication strategies aimed at replicating the intricate hierarchy observed in T/LBJs (Fig. 1).

### Electrospinning

Controllable electrodynamic droplet deposition enables the manipulation of various properties, such as size, morphology, porosity, and inclusions. A common practice is to overlay the initially prepared component and subsequently adjust the parameters for secondary electrospinning [45,73]. Moreover, a co-electrospinning device was developed for gradient generation [200]. Dual spinnerets were interconnected in parallel via wires to collect fibers and form overlapping deposits at their intersection. Of note, it is necessary to optimize the nozzle to avoid 2 streams becoming mutually exclusive. Multimaterial electrospinning was applied for advanced fabrication. Additives include minerals [55,76,115], GFs [71,74], collagen [45], metal ions [181], and varying concentrations of single polymer [75]. An alternative approach involved coaxial electrospinning with a dual inlet or outlet system. In the former scenario, 2 solutions were simultaneously electrospun at opposing flow rates to form gradient scaffolds. Two syringe pumps were connected in a “Y” shape [71,74,75] or shifted along mandrel length to adjust region-wise deposition [52]. The latter was realized by a dual drive extruder [115]. Screw selection in a time-dependent manner permitted gradient mixing of polymer with CaP particles. A promising application is the integration of microfluidic chips into electrospinning systems, facilitating quantitative blending of substrate material with bioactive components.

In light of the gradual transition of fiber arrangement in T/LBJ from uniaxial to staggered, the production of multiphasic scaffolds necessitates the implementation of technologies to control fiber orientation. The first method involves adjusting the speed of the collector rotation. In 2002, Matthews et al. [201] demonstrated that the speed of the drum significantly affects the orientation of electrospun nanofibers, giving scaffolds controllable mechanical properties. Incorporating this modification, the collector speed for the preparation of tendon fibers was set to 5,000 rpm, and for bone fibers, the collector speed was reduced to 2,000 rpm [78]. Six different collector speeds were tested, and it was ultimately determined that a speed of 0.14 cm s^−1^ was suitable for producing anisotropic yarns, while the yarns harvested at 1.09 cm s^−1^ exhibited the best isotropy [70]. The second method is to specialize collection devices, including magnet-assisting device [202], stapler-shaped metal frames [43], rotating steel cones [69], and dual-drum collectors [72]. Moreover, micropatterned electrospun fibers can impart scaffolds with wavy structures for tendon mimicry. G-code-controlled platform movement implemented a structural gradient from crimp to grid [92]. Three layers of curl fibers were stacked together with a crimp angle of 0° to 30°, a crimp length of 2 mm, and a fiber spacing of 50 to 200 μm. In contrast, the 3 layers of PCL straight fibers formed a 0° to 90° lay-down angle and a grid size of 50 to 200 μm in the grid part.

In conclusion, the flexibility and compatibility of electrospinning serve as catalysts for researchers to push the boundaries of this technique. Notably, the topological cues provided by the crimped fiber are sufficient to support T/LBJ healing, exhibiting accelerated tissue regeneration and inhibition of muscle fat infiltration in vivo [84]. After repeated stretching, fiber slippage that may occur with untreated electrospinning scaffolds, which hinder cell migration, is avoided. In fact, ethanol plasticizers can convert the residual stress of electrospun fibers into increased crystallinity (resulting in an increase in the modulus) or longitudinal shrinkage (resulting in a crimp feature) [80]. This has inspired researchers to faithfully reproduce the T/LBJ structure using this general strategy.

### 3D printing and bioprinting

**Fig. 6.**
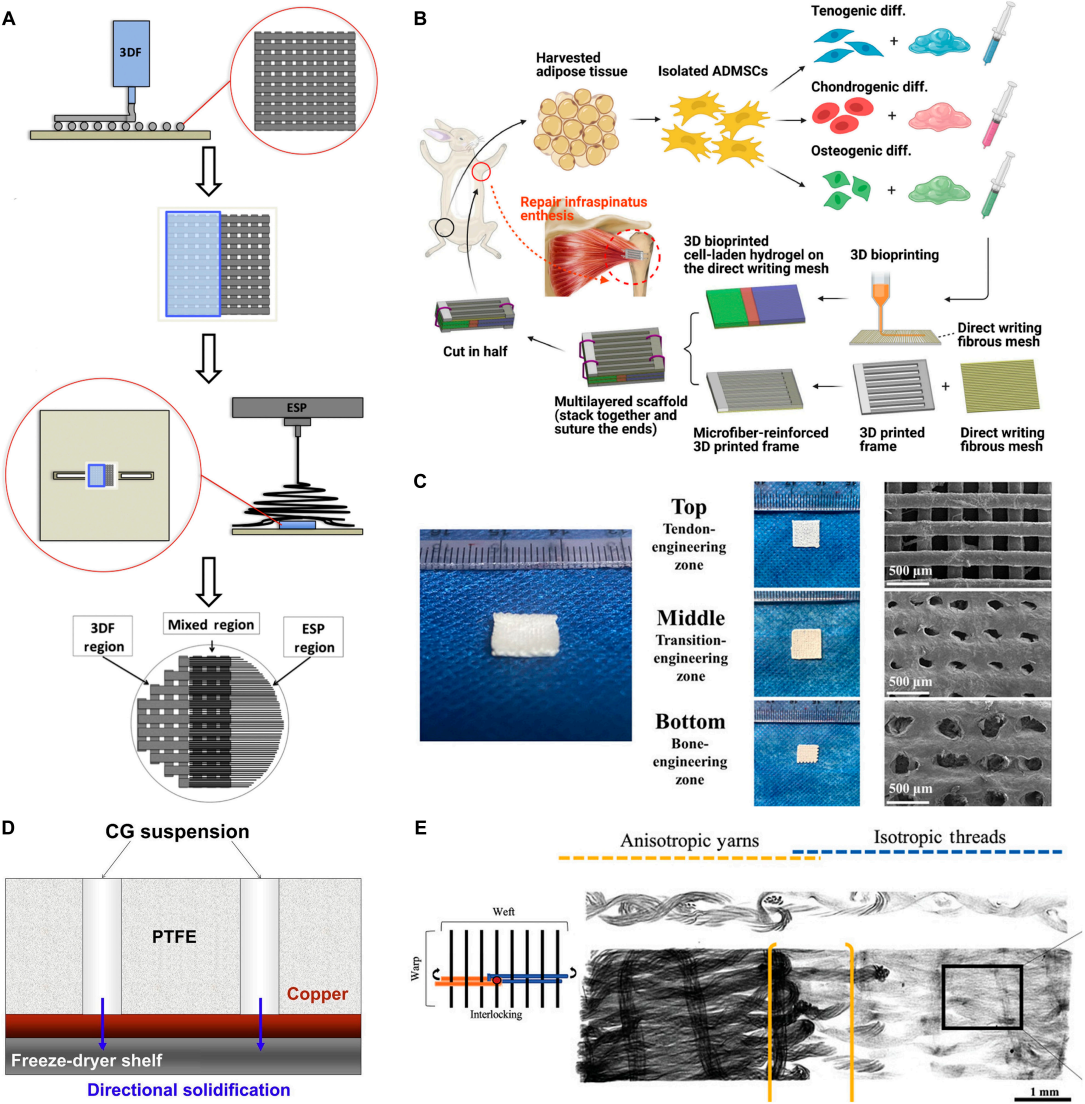
Illustrated overview of characteristic fabrication process of tendon/ligament-to-bone graded scaffolds. (A) Layer-by-layer 3D printing with a 90-degree interlamination deviation. Scaffold was then partially covered for electrospinning. Reproduced with permission [51]. Copyright 2016, IOP Publishing. (B) Fabrication process of the bioprinted multiphasic constructs with differentiated ADSCs in advance and reconstruction of rabbit infraspinatus tendon and humerus. Reproduced with permission [105]. Copyright 2022, Elsevier. (C) (i) Gross view and (ii) SEM scanning of triphasic bioprinted scaffold with increasing pore diameter from tendon to bone phase. Reproduced with permission [96]. Copyright 2023, ACS. (D) Assembly of 2 substrates with mismatched thermal conductivity for fostering anisotropic pore formation in tendon phase of the scaffolds. Reproduced with permission [220]. Copyright 2011, Elsevier. (E) Weaving process (left) and μCT imaging of biphasic fabric. Reproduced with permission [70]. Copyright 2022, Wiley.

3D printing (additive manufacturing) is a rising technology that fosters innovation in orthopedic tissue engineering [203]. At the core of this technique lies layer-by-layer printing programmed by computer-aided design software that organizes the geometric details orderly and accurately through layer-by-layer printing. The effects of printing speed and extrusion temperature on fiber orientation and tensile properties on BLB scaffold were discussed, necessitating adaptation to the characteristics of the targeted material [47]. Composition and structural gradients are intuitive changes brought by 3D printing. The micronozzle can regulate the component distribution [167,204], and porous structure of multiphasic scaffolds [98,116], or customized particular shape (such as tapered bolt) [205]. By depositing fibers layer by layer with a 90-degree shift, the bone-imitating mesh was plotted [51]. Partially obscured by paper foil, electrospun uniaxial fibers were combined in a section to complete bionic 3-phase design with transition zone (Fig. 6A).

Unfortunately, traditional 3D printing is incapable of reproducing fine structures at the interface [206]. Melt electrowriting was developed with spatial resolution at the micrometer to submicrometer level [79,207]. Due to the high temperature of the extrusion, PCL at the beginning of the scaffold was firmly fused, and then straight written as filamentous microfibers in the electrospinning process, forming a gross appearance of “fusion-uniaxial arrangement-fusion” (Fig. 6B) [105]. PCL particles were pneumatically extruded at 100 °C and collected on energized glass with planar mobility to form a stacked and adjustable wavy structure [92]. Another methodological innovation is the introduction of nondestructive Raman spectroscopy [121]. Owing to its submicrometer-scale locating precision, it was applied for generating biochemical composition maps of the enthesis.

In recent years, inkjet-based, extrusion-based, or photo-assisted 3D printing has sparked a bioengineering revolution. Based on these technologies, bioprinting employs pretreated liquid phase systems containing cells, bioactive molecules, or dECM powder as ink, and inherits the hierarchical and reproducible display of 3D printing. Currently, extrusion bioprinting is the most widely applied method, where materials are stacked into a spatiotemporally ordered 3D architecture through the force generated from feed systems such as piston, screw, or pressurized air [208–210]. The coordinated movement of the nozzle ensures shaping flexibility and versatility. Inkjet bioprinting refers to precise delivery of tiny droplets carrying biologically active substances on a mobile substrate to form specific patterns in tridimensional structures [211]. In particular, the nozzle can controllably produce droplets only when receiving the jet signal, namely, “drop-on-demand”, and exhibits greater precision and higher ink utilization efficiency [212]. Photo-assisted applications are less commonly seen in multiphasic scaffolds so far due to the absence of photosensitive bioinks.

Several strategies have been reported to establish hierarchy in bioprinted products. In a multizonal scaffold equipped with BMSCs and TSPCs, dual-channel bioprinting was adopted to enable multicellular loading [148]. Bioinks carrying different cells were transferred to 2 syringe channels of the printer, cryogenically stored at 4 °C for 20 min, followed by extrusion by air pressure at 20.5 °C at 20 to 30 kPa, layer-by-layer deposition, and photocrosslinking solidification. Similarly, a multicomponent bioprinting strategy was developed under the assistance of a microfluidic print head with multiple inlets and single outlets [213]. PLGA microspheres packaging BMP-2, TGF-β, and bFGF were prepared after two emulsifications [170], where hUCMSCs were suspended at a density of 2 × 10^6^ cells/ml. Bioinks from different syringes were extruded onto agarose substrates at an optimized flow rate of 800 l h^−1^. Three layers of GFs were stacked into constructions with a total thickness of 300 μm. In order to achieve accurate gradient, the emphasis was raised about calculating transient laminar flow stimulation before printing [214], suggesting numerical estimation as methodological iteration.

The combination of bioprinting and dECM may be the closest attempt to replicate the structure and components of native interface tissues. Major barriers include poor integrity and slow gelation of dECM bioinks. In addition, printing parameters such as speed and temperature must be individually optimized [215]. In this regard, Zhang et al. [96] successfully printed acellular matrix scaffolds for enthesis repair using a 3-stage approach, mainly by adjusting the air pressure and feeding rate during the printing process. Specifically, in the bone phase with a pore size of 300 μm, the pneumatic pressure and feed rate were set to 0.7 MPa and 10 mm s^−1^. In the fibrocartilage region, air pressure and feed velocity were reduced to 0.5 MPa and 8 mm s^−1^ to obtain a 150-μm pore size, and further downsized to 0.3 MPa and 5 mm s^−1^ in the tendon-derived ECM zone (Fig. 6C). A series of procedures was established for several multiphasic scaffold construction [145–147]. Based on characterization, PU/PCL with a 6:4 weight ratio was chosen as the frame material considering its elasticity and acceptable extrusion melting temperature range. Subsequently, BMSCs were uniformly dispersed in 3 pretreated dECM bioinks. Relying multiple nozzles to prepare micropatterns, the cell-loading bioinks were selectively deposited between the polymer layers at a pressure of 30 to 40 kPa. The layer structure was maintained at 200 to 250 μm long, 400 to 600 μm wide, and 300 to 350 μm high until achieving a thickness of 1 mm [146,147]. Apart from providing mechanical support, cell-mediated gel densification can impose uniaxial strain on stem cells [145]. Scaffolds were then incubated at 37 °C for 30 min for gelation to serve as a biomimetic environment for tendons, fibrocartilage, and bone.

Customized scaffold geography adapts to the actual condition of the injury and can be personalized for T/LBJ defect. However, mismatched heat resistance and rheological properties limit material printability, narrowing the material selection other than synthetic polymers. More in vivo studies are needed to provide compelling evidence of feasibility.

### Freeze-drying

Freeze-drying (lyophilization) is a simple, cost-effective technique, wherein crystal sublimation constitutes the fundamental step due to the creation of corresponding spaces within the scaffold [216,217]. Capable of producing porosity of approximately 90% and pore sizes ranging from 20 to 200 μm, lyophilization was proved suitable for bone-relating engineering [218]. The layered crosslinking of collagen mixture solutions was frozen and stacked together followed by lyophilizing methods to prepare a bone–fibrocartilage–tendon biomimetic scaffold [95]. Furthermore, microstructural modification can be achieved by adjusting solution concentration, freezing temperature, rate, and thermal conduction direction to obtain crystals with different shapes and sizes [219].

Several studies to achieve directional crystal formation have been reported. Devices filled with silk fibroin solution were placed in insulated polystyrene foam to produce vertically grown dendritic crystals at low temperatures, followed by sublimation [44,173]. The cooling rate was adjusted for pore size regulation. Parallel pore orientation was observed, which was subsequently used in the tendon-simulating area of the biphasic scaffold. A set of lyophilizing devices for directional coagulation was developed to create consistent pore orientation (Fig. 6D) [220]. Specifically, the mold consisted of polytetrafluoroethylene with cylindrical holes with a copper substrate attached to the bottom, and collagen–glycosaminoglycan (CG) solution filled in the holes. In the process of crystal sublimation, the bicomponent mold with significantly mismatched thermal conductivity promoted heat transfer in a single line, thereby excavating longitudinal channels. Applications of the device in tendon regeneration were reported [221,222]. By incorporating CaP into CG solution and employing conventional freeze-drying techniques, an isotropic bone mimicry structure was constructed. The 2 were then crosslinked as a heterogeneous scaffold, containing compartments with varying structures and mineral content, to observe the tri-lineage differentiation of MSCs in enthesis repair [68,223].

A huge challenge in these applications is the necessity to individually produce the different parts of the scaffold for final assembly. However, crosslinking alone does not guarantee sufficient mechanical strength, which can interfere in vivo implantation. To address this issue, a modification is proposed by introducing a polyethylene glycol hydrogel at the interface, with the help of gelling kinetics to integrate the CG and CG-CaP region, providing excellent biomechanical properties, and was expected for tendon/ligament–bone insertion repair [114]. Further, gradient structures can also provide tighter connections. Peniche Silva et al. [59] prepared biphasic scaffolds with mutant and smooth phase transitions by freeze-drying and salt leaching. Directional solidification coworked with liquid-phase cosynthesis to achieve gradient distribution at the pore structure and mineralization level. To be specific, a layered CG-CaP suspension was added to the CG solution in the mold as described above and automatically mineralized in gradient through particle permeation, which is subsequently freeze-dried [99].

### Textiling

The step-by-step assembly from fiber to yarn to fabric permitted precise adjustment of size and proportions to provide a hybrid helix [224]. Thus, knitting, weaving, and braiding process have incomparable merits in the representation of intricate and delicate structures [225]. By adjusting parameters such as stitch length, stitch angle, and duplication shift, textile processes can precisely produce multizonal micropatterns. In bone–ligament cell coculture systems [86], zigzag needle movement was used in the ligament phase to form a wavy architecture, distinct from the triaxial line arrangement of the bone compartment. Drawing inspiration from the synovial-wrapped fiber bundles, the core–shell fabric was designed to obtain outstanding axial tensile strength [199]. Braiding technique was applied to arrange numerous combinations between shell and fiber bundle, exploring optimal construction of mechanical properties.

In addition to endowing fibers with unique features, the textiling process can also controllably assemble flat geometries into spatial structures. An advanced textile platform was developed to complete compositional gradient [196]. PCL was hybridized with gelatin and HAp, respectively, to simulate collagen-rich tendon and highly mineralized bone. Microfibers were acquired through customized wet-spun device. At a constant flow rate, liquid-phase polymer was injected into a supporting coagulation bath, instantaneously generating superfine fibers and being captured by the collector. Tests of different solutions and flow rates indicated that the conjugation of HAp and extrusion rates can affect fiber diameter and orientation, highlighting the simplicity and adjustability of the platform. By crocheting (a weaving technique), filaments mimicking tendons and bones were assembled into 3D scaffolds, demonstrating spatial regulation over the multilineage fate of ADSCs.

In the microstructure of ligaments, fiber bundles are enveloped by the synovial membrane, and the simplified physical model is depicted as a shell filled with woven bundles. This intricate structure is replicated through a weaving process to create a “core–shell” texture with internal stability, excellent strength, and stiffness [199]. By combining hydrothermal powder preparation with thermally induced phase separation, a more sophisticated structure was achieved [226]. Briefly, the electrospun double-layer yarns were wound into braid-style artificial ligaments, with each yarn consisting of silk fibroin microfibers as the core and being wrapped in porous poly(l-lactide-co-ε-caprolactone) (PLCL). The mesoporous HAp powder was blended with polylactic acid (PLA) and then cast at both ends of the fabric. This process initiated phase separation and solidified the polymer after segmented refrigeration. Macroscopically, it presented a BLB structure with 2 mineralized bone phases connected by tough artificial ligaments.

In other studies, textile processes were employed to fabricate multiphasic scaffolds with exceptional tensile properties. Parallel filaments were twisted into S-shape configuration and integrated into 3D printed blocks for hard-to-soft biomimetic synthesis [91]. Bundles of electrospun fibers, doped or undoped with HAp, were twisted into isotropic and anisotropic yarns [70], which were subsequently placed longitudinally and transversely at a spacing of 0.5 cm, respectively. The warp and weft were interlocked into topographically and compositionally continuous network (Fig. 6E). Using a custom separator, a macro-porous scaffold, embroidered by sericin-extracted silk, was divided into 3 partitions while maintaining its structural integrity. These partitions served as carriers for lentiviral carriage, coding GF-related genes [128], and coating of the ECM substances [227].

## Challenges and Prospectives

Multiphasic scaffolds exhibit potential to further expand their applications in T/LBJ healing. Creative and practical scaffold characteristics and preparation methods have emerged, inspiring subsequent research. However, there are still many shortcomings in the current researches, and we summarize the challenges in this field and provide ideas for improvement based on the latest progress.

In addition to biological activity, tissue engineering for T/LBJ healing needs to meet some basic properties, such as degradability, mechanical strength, and cytocompatibility. PCL, a commonly used electrospinning material, degrades slowly in the human body, which affects the penetration of regenerated tissues in the late stage. The acidic environment generated by polyester materials representing PLGA during the degradation process may interfere with cell growth and aggravate the inflammatory response. In addition, in most of the bi-/tri-/quadra-phasic scaffold, there is a lack of direct evidence to demonstrate the mechanical stability of interlaminar adhesion, which is essential for long-term therapy. Also, few convincing study is reported on whether the inhomogeneous distribution of fiber morphology and pore distribution increases the possibility of scaffold collapse or not.

Fibrocartilage is an important transition component in the structure of T/LBJ, and although it has gradually received attention, some studies still ignore the verification of cartilage layer in tissue staining. Restoration of calcified and noncalcified stratification appears to be necessary to further guarantee a smooth transition of mineralization and mechanical conduction. Notably, most studies confuse chondrocyte populations; however, there are differences in cell morphology, developmental stages, and phenotypes within them. For example, hypertrophic chondrocytes in the calcified layer exhibit higher metabolic rates and express more genes related to endochondral ossification including angiogenesis factors and MMP, while chondrocytes in the noncalcified layer are more related to cartilage matrix synthesis, such as Col II and proteoglycans. Whether the subtypes of chondrocytes in T/LBJ play an equally important role as in osteochondral structure deserves the attention of follow-up researchers. Moreover, most of the verification of therapeutic efficacy has focused on tissue-specific marker expression, micro-stereoscopic imaging, histochemistry, and mechanical testing. The lack of exploration at the molecular biology level seems significant. T/LBJ healing involves crosstalk between osteogenic, chondrogenic, and tenogenic pathways, and inadequate research focuses on how signaling transductions cooperate with each other.

Although inorganic materials received attention from the beginning, the primary focus rarely shifted away from CaP and its isomers. Simple methods have been reported to achieve gradient HAp distribution by gradual immersion in SBF, but only surface mineralization can be ensured, and the calcification process cannot be precisely controlled. The manipulation of magnetic fields or gravity in osteochondral regeneration scaffolds would be refreshing for these researchers [228,229]. Recently, the status of other inorganic substances, such as metal ions and BGs, has gradually risen. Zn^2+^ and Cu^2+^ received the most focus, but the potential connection between the gradual release of metal ions and the therapeutic repair of the enthesis is still unclear. Furthermore, in multiphasic engineering, the development of strontium ions is still underway, particularly in relation to the simultaneous promotion of Sr^2+^ on MSC osteogenesis and chondrogenesis, as well as macrophage M2 polarization [230].

Organism-derived multiphasic scaffolds are infused with young blood due to their unparalleled biocompatibility, degradability, and bioinducibility. Whether in terms of smooth transition or component diversity, dECM stands out uniquely. However, balancing the retention of bioactive ingredients and clearing immunogenicity appears to be a major issue. Methods for antagonizing the classical immune response in tendon/ligament–bone dECM were reported [231]. Bioprinting reconfigures the spatial arrangement of cells and/or ECMs to achieve precise phenotypic delivery. The concept of combining organ-on-chip technology with bioprinting has been proposed as a potential development for the next-generation osteotendinous platform [232]. Cell sheets have shown promising potential to complement more advanced decellularized scaffolds, particularly book-shaped designs [143], as the 2D slits between the pages favor cell sheet formation.

Mechanobiology researches had unveiled that mechanically sensitive bone and tendon ECM create distinct force-conducting environment. Stiff attribute of the matrix ranks among the influential factors in stem cell differentiation. Although disaccord remained in the range of modulus, the bone-promoting effect of the higher modulus substrate was confirmed in multiple studies [233–235]. Interestingly, substrate modulus close to physiological situations [236,237], and significantly lower (below 100 kPa) [185,238], was thought to contribute to tendon commitment. Recently, under thermal assistance, Ye et al. [239] prepared a batch of matrices with a wide elastic modulus span (about 870 to 2,670 MPa) and investigated the ascending and then descending trend of tenogenicity along with stiffness increase, which reached its peak at about 1,430 MPa. Transcriptomic evidence was served for optimal tendon stromal cell differentiation at 35 kPa [240]. Based on the data listed above, the establishment of biomimetic scaffolds with gradient substrate tensile properties has outstanding research value in tendon/ligament–bone tissue engineering. Establishing biomimetic scaffolds with gradient mechanical environment provides excellent models to uncover the threshold adaptive to tendon and bone regeneration, as accurate healing guidance.

Emerging electrical stimulation strategies show potential to promote tendon/ligament–bone integration. Electroactive materials not only modulated the elongated cell morphology and pseudopodium extension, which is necessary for tenocytes, but also promoted osteogenesis [241,242]. Notably, the coupling of mechanical and electrical stimuli, as exemplified by piezoelectric effects, has garnered attention in bone, cartilage, and tendon engineering [243–245]. Gradient arrangement of piezoelectric nanofibers promotes cell homing to suitable positions under ultrasound excitation and improved cell differentiation and tissue regeneration [246]. These findings shed light on novel avenues for multiphasic osteotendinous interface design.

Navigating the multidimensional intricacies of tendon-to-bone healing will continue to be focused in future research. Electrical cues have been shown to orchestrate a myriad of factors, including cellular orientation, mechanical integrity, and cargo distribution [247]. Recent strategies feature dual gradients of pore size and fiber orientation, enhanced by asymmetric modification with bFGF [248]. These innovations are highly promising for interfacial tissue engineering. Ternary functional modeling and microscopic examination provided references on the network of mineralization, fiber arrangement, and mechanics [249,250]. Such data are poised to spur further investigations into the intrinsic relationships among different gradients, thereby enabling the development of more physiologically relevant, biomimetic simulations.

## Conclusion

In conclusion, there is growing interest in the multiphase engineering of tendon/ligament–bone reconstruction, and several innovative and effective strategies have emerged to offer promising prospects for patients with RC tears and ACL ruptures. We have summarized the multiphase strategies that have been developed to promote tendon/ligament–bone healing. Additionally, we have introduced applications on substrate materials, layout, characteristics, and preparation methods. We have also evaluated the existing exploration and technologies to provide new insights for in-depth development. Based on progress and potential defects, we further analyzed the current situation in tendon/ligament–bone multiphasic scaffolds. Future microstructure elucidation and biomaterial development are expected to encounter challenges and push the boundaries of interdisciplinary fields.
